# Integrating enzymology and microbial ecology in food fermentation: Molecular mechanisms, metabolic interactions, and future perspectives

**DOI:** 10.1016/j.crfs.2026.101609

**Published:** 2026-10-26

**Authors:** Jing Hao, Shengqi Zhang, Feng Xiong, Nan Wu, Mokhtar Dabbour, Benjamin Kumah Mintah, Chunhua Dai, Rong Zhang, Zhaoli Zhang, Haile Ma, Ronghai He

**Affiliations:** a School of Food Science and Engineering, Jiangsu University, 301 Xuefu Road, Zhenjiang, Jiangsu, 212013, China; b Institute of Food Physical Processing, Jiangsu University, 301 Xuefu Road, Zhenjiang, Jiangsu, 212013, China; c Department of Agricultural and Biosystems Engineering, Faculty of Agriculture, Benha University, P.O. Box 13736, Moshtohor, Qaluobia, Egypt; d CSIR – Food Research Institute, P.O. Box M20, Accra, Ghana; e Department of Agro-processing Technology and Food Bio-sciences, CSIR College of Science and Technology (CCST), Accra, Ghana; f School of Food Science and Engineering, Yangzhou University, 196 Huayang West Road, Yangzhou, Jiangsu, 225127, China

**Keywords:** Fermented foods, Enzyme–microbial interactions, Fermentation ecosystems, Multi-omics, Synthetic microbial consortia, Precision fermentation

## Abstract

Food fermentation is a widely applied biotechnological process that improves the nutritional, sensory, and functional properties of foods through coordinated biochemical and microbial transformations. Although fermentation optimization has traditionally focused on microbial strain selection and process regulation, fermentation outcomes cannot be adequately explained by microbial composition alone, but instead emerge from the coupling among microbial functions, enzyme activities, substrate properties, environmental conditions, and metabolic networks. This review develops an enzyme–microbial interaction framework, herein discussed in the context of enzyme–microbial synergistic fermentation systems, to integrate enzymatic substrate transformation with microbial ecological and metabolic responses. The molecular mechanisms underlying these interactions are discussed, including substrate depolymerization, carbon and nitrogen redistribution, microbial cross-feeding, ecological interactions, enzyme regulation, and environmental niche adaptation. Particular emphasis is placed on the extent to which enzyme-mediated changes in substrate accessibility reshape resource availability and thereby influence microbial succession, pathway utilization, metabolic flux distribution, and the formation of flavor- and function-associated metabolites. Furthermore, the capabilities and limitations of multi-omics and computational approaches for resolving enzyme–microbial interactions are critically evaluated, particularly with respect to distinguishing functional potential from active biochemical processes, establishing causal relationships, improving quantitative prediction, and facilitating industrial translation. By integrating enzymology, microbial ecology, and systems-level analysis, this review provides a mechanism-oriented framework that links substrate accessibility, ecological organization, metabolic allocation, and fermentation phenotype, and highlights future directions toward more predictable, controllable, and sustainable fermentation strategies.

## Highlights

• Enzyme-microbial synergy governs fermentation performance.
• Extracellular enzymes reshape microbial resource utilization.
• Microbial interactions drive metabolic redistribution during fermentation.
• Mechanistic integration supports predictable fermentation design.

## Graphical abstract

**Figure:**
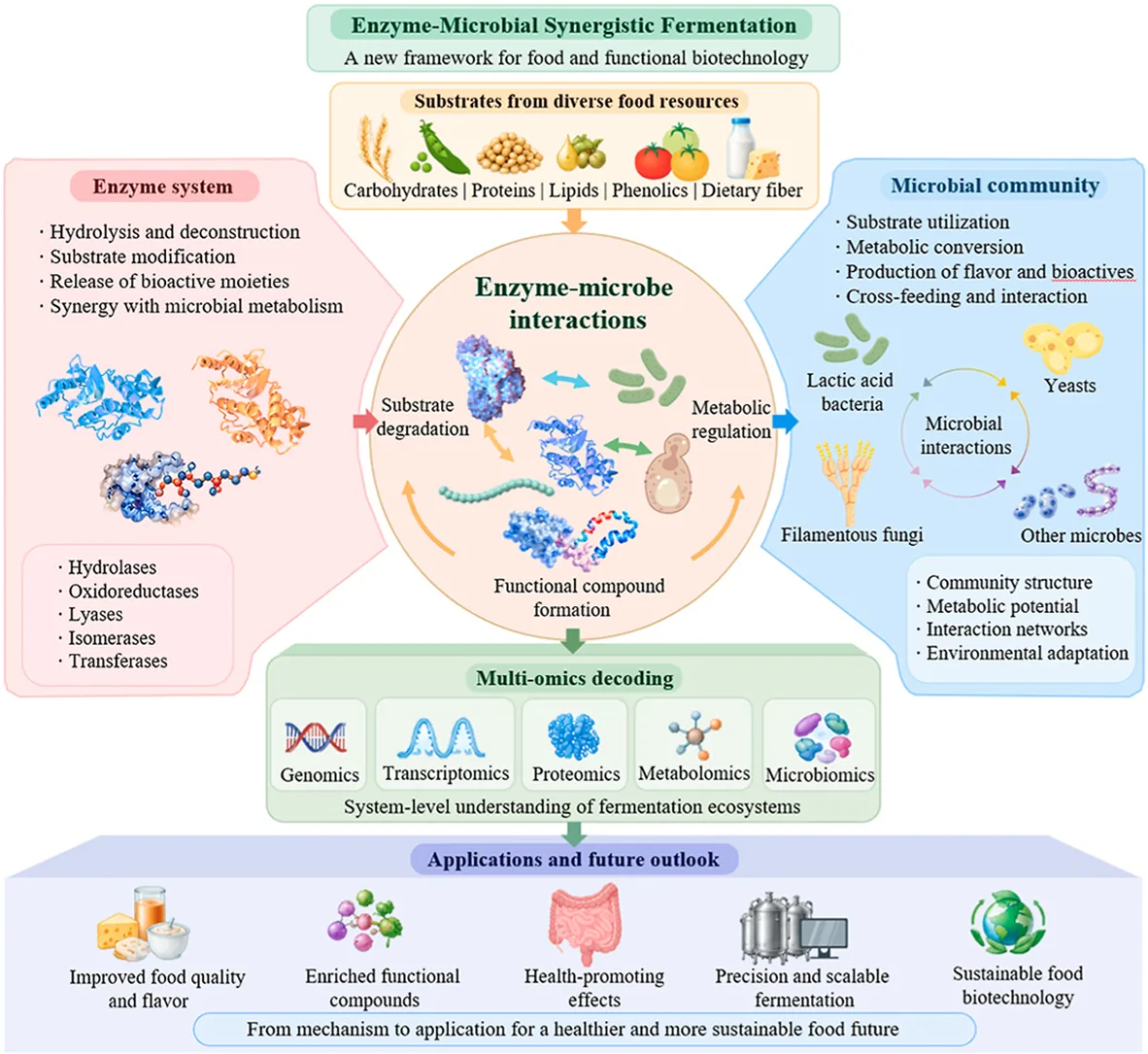

## 1 Introduction

Food fermentation is an ancient and widely applied biotechnological process that improves food preservation, sensory properties, nutritional value, and functional characteristics. It is extensively applied in the production of dairy products, cereal-based foods, soy-derived products, beverages, and other fermented foods (Wolfe and Dutton, 2015; Farag et al., 2020; Wang et al., 2021a,b; Yin et al., 2020). During fermentation, microorganisms mediate diverse biochemical transformations, including carbohydrate utilization, protein degradation, organic acid production, volatile compound formation, and generation of bioactive metabolites, thereby influencing the quality and functionality of fermented products (Dai et al., 2017; Yin et al., 2020; Zhang et al., 2016). Traditionally, fermentation optimization has primarily relied on microbial strain selection, process parameter adjustment, and environmental control. However, increasing evidence suggests that fermentation performance is influenced not only by microbial composition but also by interactions among microbial communities, enzyme activities, substrate characteristics, and metabolic networks (Wolfe and Dutton, 2015; Xin and Qiao, 2025; Nikoloudaki et al., 2024). Accordingly, fermentation research is increasingly shifting from a microorganism-centered perspective toward an ecosystem-level understanding, in which microbial functions, enzymatic activities, substrate transformation, and environmental regulation are considered interconnected determinants of fermentation performance.

Within fermentation ecosystems, enzymes function as catalytic determinants of substrate accessibility, while their ecological effects are mediated through changes in nutrient availability and subsequent microbial metabolic interactions. Microbial-derived enzymes contribute to the degradation of complex macromolecules, generating fermentable sugars, peptides, amino acids, and other intermediates that support microbial growth and metabolite formation (Christensen et al., 2022; Zhao et al., 2015). Conversely, exogenous enzyme supplementation can modify substrate structures and alter biochemical conditions available for microbial metabolism. For example, cellulase-assisted lactic-acetic acid fermentation of jujube puree modified physicochemical characteristics, phytochemical composition, volatile profiles, and antioxidant capacity (Boasiako et al., 2024a,b). Similarly, microbial proteolytic activities during soybean meal fermentation promoted protein hydrolysis and peptide formation, improving nutritional characteristics (Liu et al., 2023). These observations suggest that enzymes influence fermentation not only through substrate conversion but also may indirectly influence microbial interactions by altering substrate accessibility and nutrient availability. Recent reviews have substantially advanced our understanding of microbial community dynamics, synthetic microbial consortia, fermentation-associated multi-omics characterization, and artificial intelligence-assisted process optimization (Nikoloudaki et al., 2024; Huang et al., 2025; He et al., 2026). However, many existing studies have emphasized specific components of fermentation systems, including microbial composition, community engineering, analytical technologies, or process optimization, whereas the mechanistic integration of enzyme-mediated substrate transformation with microbial ecological regulation remains comparatively limited. Therefore, this review adopts enzyme–microbial synergistic fermentation systems as an organizing framework to connect extracellular enzymatic functions, microbial interactions, metabolic flux redistribution, and fermentation outcome. The comparative scope and distinctive contribution of this review relative to previous reviews are summarized in Table 1.

**Table 1:** Comparison between previous reviews and the present review on enzyme–microbial synergistic fermentation systems.

Previous review	Main research focus	Coverage of enzyme–microbial integration	Aspects insufficiently covered	Distinctive contribution of the present review
Wolfe and Dutton (2015)	Fermented foods as experimentally tractable microbial ecosystems	Introduced microbial ecology and community interactions in fermented foods	Enzyme-mediated substrate transformation was considered mainly as a biochemical process rather than an ecological driver shaping microbial interactions and fermentation trajectories	Establishes an enzyme–microbial synergistic fermentation framework explicitly linking substrate accessibility, microbial ecology, metabolic allocation, and fermentation outcomes
Jin et al. (2024)	Synthetic microbial communities (SynComs) for fermented foods	Summarized strategies for consortium construction, stability, and functional design	The role of extracellular enzymes in regulating resource accessibility and microbial assembly received comparatively limited attention	Extends SynCom research by incorporating enzyme-mediated resource accessibility and substrate transformation as mechanistic determinants of community assembly and fermentation performance
Yuan et al. (2025)	LAB–yeast interactions, multi-omics, and artificial intelligence	Reviewed microbial interactions and computational approaches for fermentation analysis	Enzymatic regulation of substrate conversion was insufficiently integrated with microbial ecological interactions and metabolic organization	Integrates extracellular enzymatic processes with microbial ecological interactions to explain metabolic coordination during fermentation
Xin and Qiao (2025)	Synthetic biology, metabolic engineering, and microbial consortia	Focused primarily on engineering microbial systems for functional optimization	Endogenous microbial enzymes and exogenous enzyme-assisted substrate transformation were not incorporated into a unified fermentation framework	Expands microbial engineering concepts by integrating endogenous enzyme production, exogenous enzymatic intervention, microbial ecology, and metabolic regulation into a unified conceptual framework
He et al. (2026)	Machine-learning-assisted microbial community design	Reviewed AI-assisted prediction and optimization of microbial communities	Biological interpretation remained largely disconnected from experimentally supported enzyme-mediated mechanisms	Places artificial intelligence within a mechanism-oriented enzyme–microbial framework, emphasizing biological interpretation, causal inference, and experimentally supported prediction
Karimi et al. (2026)	Microbiome metabolic modeling in fermented foods	Reviewed computational prediction of microbial metabolism and community behavior	Computational predictions were only weakly connected with experimentally validated enzymatic processes and biochemical mechanisms	Integrates enzymology, microbial ecology, metabolic modeling, and systems biology to bridge molecular mechanisms with predictive fermentation models
Zhang et al. (2026a)	Omics-based strategies for reproducible fermentation	Reviewed multi-omics characterization and standardization of fermentation systems	Enzyme activity was primarily treated as one functional outcome rather than a dynamic regulator of microbial ecological processes	Provides a mechanism-oriented synthesis integrating enzyme activities, microbial ecology, multi-omics evidence, computational modeling, and industrial translation from molecular mechanisms to process optimization

To address this gap, this review proposes an integrated framework of enzyme–microbial synergistic fermentation systems, in which endogenous microbial enzyme production and activity, exogenous enzyme intervention, microbial ecological interactions, and metabolic regulation are considered interconnected processes shaping fermentation trajectories. Within this framework, microbial-derived enzymes, exogenous enzymes, microbial communities, metabolic pathways, and regulatory networks are regarded as interconnected components that collectively influence substrate conversion, metabolic flux distribution, and product formation. Specifically, enzyme-mediated substrate transformation regulates resource accessibility, microbial interactions influence resource utilization and metabolic exchange, and the resulting biochemical outputs provide measurable indicators for evaluating fermentation performance and guiding process control (Fig. 1).

**Fig. 1:**
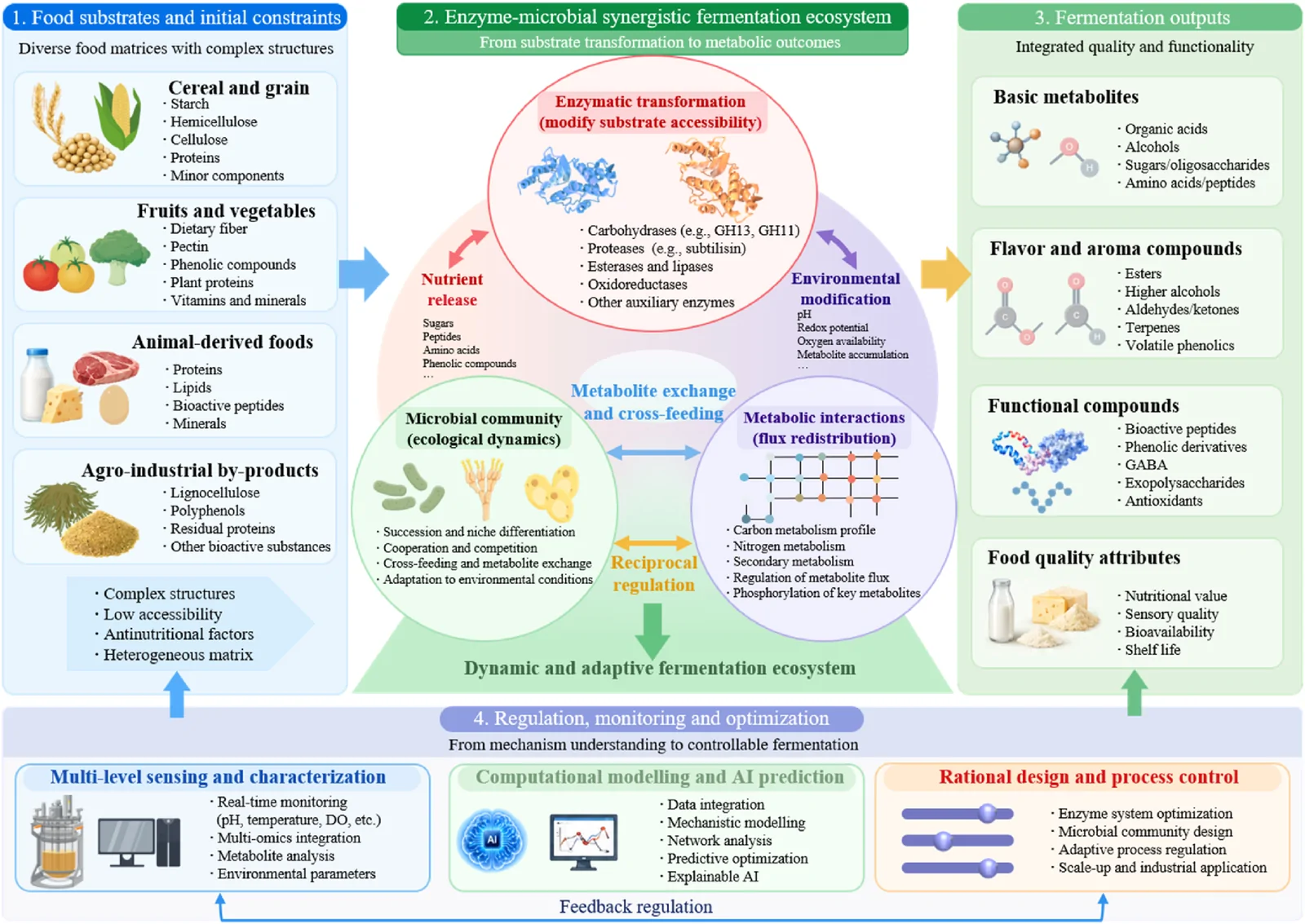
Conceptual framework of enzyme–microbial synergistic fermentation systems integrating microbial ecology, enzymatic transformation, metabolic interactions, and fermentation control.

Accordingly, this review summarizes recent advances in enzyme–microbial interactions and discusses their contributions to substrate transformation, microbial ecological dynamics, metabolic regulation, and fermentation optimization. Within this framework, enzyme–microbial synergistic fermentation systems are considered as multi-layered regulatory networks consisting of five interconnected components: enzyme-mediated substrate transformation, microbial resource utilization, ecological interaction and adaptation, metabolic pathway redistribution, and measurable fermentation outcomes. These components form a sequential yet feedback-regulated process linking molecular events with fermentation phenotypes. Rather than organizing current knowledge according to individual fermented food categories, this review follows a mechanistic framework consisting of extracellular substrate transformation, microbial cooperation and competition, metabolic cross-feeding, regulatory responses, environmental adaptation, and system-level prediction. Their consequences for carbon and nitrogen redistribution, flavor formation, and functional metabolite production are further discussed. In addition, recent advances and limitations of multi-omics and computational approaches for resolving enzyme–microbial interactions are critically evaluated, with emphasis on causal interpretation, quantitative prediction, and industrial translation.

This review focuses on food fermentation systems in which enzyme activities and microbial ecological processes interact to influence fermentation outcomes. Studies involving purified enzymes, individual strains, defined microbial consortia, and complex food matrices are considered to provide different levels of mechanistic evidence. By integrating enzymology, microbial ecology, and computational approaches, this review aims to clarify the molecular basis of enzyme–microbial interactions and provide a conceptual foundation for the rational design of more predictable fermentation systems.

## 2 Molecular mechanisms underlying enzyme–microbial interactions in fermentation ecosystems

Enzyme–microbial interactions represent an important mechanistic basis through which biochemical transformation, microbial ecological dynamics, and metabolic adaptation are integrated during food fermentation. Rather than functioning solely as isolated catalytic reactions, enzyme activities can be coupled with microbial community assembly, substrate utilization, metabolite exchange, and environmental adaptation. During fermentation, microorganisms contribute to biochemical conversion through both metabolite production and enzyme secretion, whereas enzyme-mediated modification of food matrices alters resource accessibility and subsequently influences microbial ecological processes. Therefore, enzyme–microbial interactions should be considered as dynamic regulatory processes in which enzymatic activities influence substrate availability, while microbial metabolic activities and environmental conditions further regulate enzyme expression, activity, and functional outcomes. These interactions can be broadly understood through several interconnected modes, including extracellular substrate liberation, metabolic cross-feeding and resource exchange, ecological cooperation and competition, resource-mediated niche modification, and regulatory control of enzyme expression (Fig. 2). However, the evidence supporting these interaction modes varies substantially among experimental systems. Studies based on purified enzymes or single strains provide direct evidence of catalytic functions, whereas defined co-culture systems and complex food fermentations provide increasing ecological relevance but involve additional biological variables that complicate causal interpretation.

**Fig. 2:**
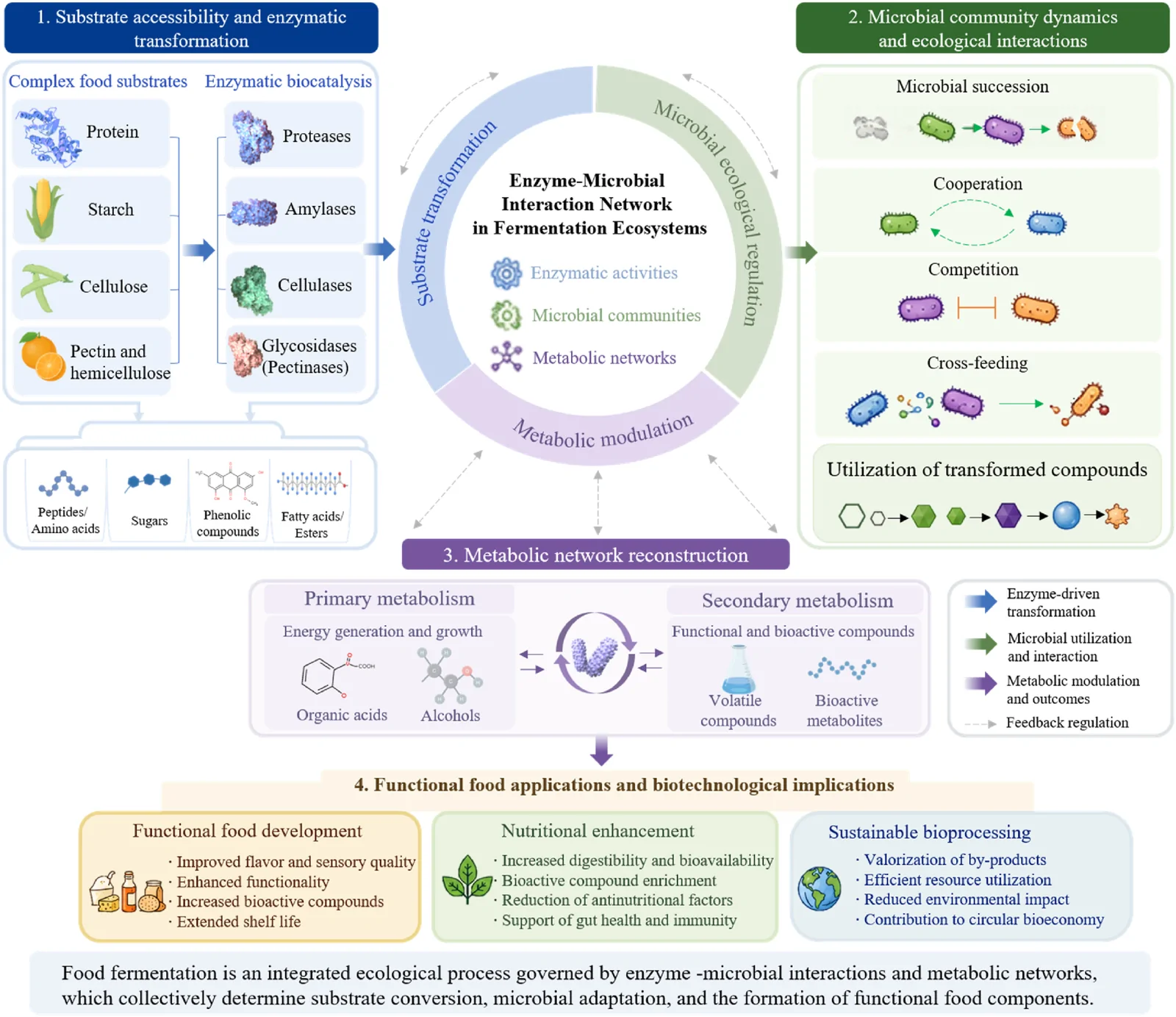
Molecular mechanisms underlying enzyme–microbial interactions during substrate transformation and metabolic regulation.

Microbial-derived enzymes and exogenous enzymes contribute differently but complementarily to fermentation processes. Microbial-derived enzymes are produced in response to substrate availability, nutritional conditions, and regulatory signals, allowing microorganisms to adapt their metabolic activities to changing fermentation environments. In contrast, exogenous enzyme supplementation provides targeted modification of food matrices and can alter the availability of carbon and nitrogen resources before or during microbial fermentation. The functional consequences of both enzyme sources depend not only on catalytic efficiency but also on whether the released substrates or intermediates can be effectively utilized by microbial communities and incorporated into downstream metabolic pathways. Representative enzyme systems involved in food fermentation are summarized in Table 2.

**Table 2:** Representative enzyme-related systems and evidence supporting enzyme–microbial interactions in food fermentation.

Enzyme system	Representative microorganisms/enzyme sources	Substrate and fermentation system	Functional contribution	Evidence context/level	Evidence and interpretation	References
Carbohydrate-active enzyme (CAZyme) systems	*Lactiplantibacillus plantarum* and other LAB	Plant-derived carbohydrates; fermented vegetables, dairy- and cereal-associated systems	CAZyme-related genes contribute to carbohydrate utilization potential by enabling degradation and transformation of complex carbohydrates	Strain-level genomic/functional evidence	Genome-based evidence identifies functional potential; enzyme activity and ecological effects require biochemical and community-level validation	Liang et al. (2023)
CAZyme-associated carbohydrate utilization	*Leuconostoc citreum* SH12	Plant-based fermentation substrates	Genomic analysis revealed diverse carbohydrate-active enzyme families associated with potential carbohydrate metabolism capacity	Genomic + phenotypic strain evidence	Supports carbohydrate-utilization capacity but does not directly establish in situ enzyme activity during complex fermentation	Dai et al. (2024)
Extracellular protease systems	*Bacillus subtilis* and other proteolytic microorganisms	Soybean meal; solid-state fermentation	Protease production promotes protein hydrolysis and generation of peptides and amino acid derivatives available for microbial metabolism	Complex food-matrix fermentation with direct enzyme/product measurements	Peptide yield reached 220.93 mg/g, protease activity was 103.37–241.93 U/g, and trypsin inhibitor activity decreased by 76.11% under enhanced SSF	(Dai et al., 2017; Liu et al., 2024)
Protease-assisted plant-protein transformation	Protease-producing microbial consortia	Rapeseed meal, soybean meal, and other protein-rich agricultural substrates	Proteolytic degradation improves substrate accessibility and promotes peptide formation during fermentation	Depending on experimental design	Evidence ranges from direct biochemical hydrolysis to microbial fermentation; ecological consequences should be interpreted only where microbial responses were measured	(Dai et al., 2022a,b; Hou et al., 2019)
Cellulase-assisted microbial fermentation	Cellulase combined with *Lactiplantibacillus plantarum* and *Acetobacter pasteurianus*	Jujube puree fermentation	Cellulase pretreatment modifies plant cell wall components and improves accessibility of fermentation substrates	Enzyme-assisted fermentation in a real food matrix	Changes were observed in physicochemical properties, phytochemical profiles, volatiles, and antioxidant-associated measurements; the relative contributions of enzyme treatment and microbial metabolism remain partially unresolved	Boasiako et al. (2024a)
Enzyme-intensified microbial consortia	CAZyme-rich microbial consortia	Corncob and lignocellulosic biomass conversion	Enzyme-assisted microbial systems enhance degradation of structural carbohydrates and improve bioconversion efficiency	Defined/mixed consortium in complex biomass systems	Supports functional coupling between enzyme-related degradation and microbial conversion; community-level causality requires controlled reconstruction	Geng et al. (2024)
Ultrasound-assisted enzymatic hydrolysis	Food-grade proteases combined with physical pretreatment	Rapeseed and other plant proteins	Ultrasound pretreatment improves enzymatic hydrolysis efficiency and modifies peptide composition	Supporting biochemical evidence	Provides mechanistic evidence for substrate accessibility and peptide generation but does not independently demonstrate enzyme–microbial ecological interaction	(Jin et al., 2016; Liang et al., 2017)
Enzymatic generation of bioactive peptide fractions	Purified or food-grade protease systems	Rapeseed meal and food-protein substrates	Enzymatic hydrolysis generates peptide fractions with antioxidant or other functional properties	Supporting biochemical evidence	Functional properties were evaluated after hydrolysis; microbial ecological effects were not directly assessed	Zhou et al. (2016)

### 2.1 Microbial-derived enzyme systems as functional drivers of substrate transformation

Microbial-derived enzymes represent important functional traits linking microbial enzymatic capacity with substrate transformation and downstream metabolite production in fermentation ecosystems. During fermentation, microorganisms contribute not only through metabolite production but also through secretion of extracellular enzymes that regulate the accessibility and conversion of complex food substrates. Unlike purified enzyme systems operating under controlled conditions, microbial enzymes function within dynamic ecological environments, where substrate composition, physicochemical conditions, microbial physiology, and community interactions may influence enzyme production and catalytic activity. Therefore, the functional contribution of microorganisms should be evaluated according to their enzymatic capacity and metabolic potential rather than relying solely on taxonomic identity or relative abundance.

The biochemical transformation capacity of fermentation systems is strongly influenced by substrate-specific enzyme repertoires that regulate the accessibility of carbon- and nitrogen-containing compounds. In carbohydrate-rich fermentation systems, carbohydrate-active enzymes (CAZymes) generally function as complementary catalytic networks rather than through a single hydrolytic step. Glycoside hydrolases, carbohydrate esterases, and related enzymes can progressively deconstruct starch- and plant cell wall-derived polysaccharides, generating soluble carbohydrates and releasing matrix-associated compounds that become available for subsequent microbial utilization. In protein-rich fermentation systems, extracellular proteases initiate macromolecular hydrolysis, followed by further peptide degradation and amino-acid release through downstream peptidase activities. Consequently, the ecological significance of these enzyme systems lies not only in bulk substrate degradation but also in their capacity to determine the composition, timing, and accessibility of resource pools available to different microbial populations.

Recent studies provide evidence that microbial enzymatic capacity is associated with differences in substrate conversion and fermentation performance. *Lactiplantibacillus plantarum* strain 84-3 was reported to possess an abundant CAZyme repertoire, including GH1, GH13, GT2, and GT4 families, together with enrichment of carbohydrate- and butanoate-related functions, supporting its predicted capacity for complex carbohydrate utilization and flavor-associated metabolism (Liang et al., 2023). Similarly, *Leuconostoc citreum* SH12 contained 193 carbohydrate-metabolism-related genes, including 59 CAZyme genes, and exhibited experimentally distinguishable utilization of multiple carbon sources, with efficient use of glucose, sucrose, fructose, and maltose and lower utilization of xylose, arabinose, mannose, and cellobiose (Dai et al., 2024). In soybean meal fermentation, *Bacillus subtilis* strain SBM_1 was associated with enhanced proteolytic activity, increased peptide accumulation, and substantial reduction of trypsin inhibitor activity relative to natural fermentation (Liu et al., 2024). Under optimized enhanced solid-state fermentation conditions, the peptide yield reached 220.93 mg/g, corresponding to a 47.74% increase relative to natural fermentation; protease activity remained within 103.37-241.93 U/g, while trypsin inhibitor activity decreased by 76.11%. In addition, peptides with molecular weights below 3 kDa accounted for 71.64% of the final peptide fraction. These quantitative outcomes provide a direct link between microbial proteolytic activity, nitrogen-resource redistribution, and measurable changes in fermentation products. Nevertheless, such observations primarily establish biochemical and process-level effects and do not by themselves demonstrate that protease activity directly caused restructuring of the microbial community. Together, these studies show that microbial enzyme repertoires and measured catalytic activities can be linked to substrate conversion outcomes; however, genomic annotation, enzyme activity, and ecological function should be distinguished as different levels of evidence.

Microbial enzyme production and catalytic activity are highly responsive to environmental conditions and microbial community dynamics (Christensen et al., 2022; Zhao et al., 2015). In complex fermentation systems, particularly solid-state fermentation (SSF), microorganisms experience heterogeneous microenvironments characterized by differences in moisture, oxygen availability, nutrient distribution, and temperature. These spatial and temporal gradients can alter both microbial population structure and enzymatic performance, making it difficult to separate ecological succession from biochemical transformation. In soybean meal fermentation, changes in bacterial composition occurred together with peptide accumulation and broader metabolic variation (Liu et al., 2023), whereas thermophilic SSF of rapeseed meal showed substantial microbial community restructuring under changing fermentation conditions (Hou et al., 2019). Such studies establish temporal and statistical associations between microbial succession and biochemical transformation, but they generally do not demonstrate that changes in a specific enzyme activity are sufficient to cause community restructuring. Quantitative relationships among enzyme abundance or activity, substrate conversion rate, microbial population responses, and metabolic redistribution therefore remain insufficiently resolved in complex food matrices.

### 2.2 Exogenous enzyme-mediated modulation of fermentation processes

Exogenous enzyme supplementation represents an intervention strategy for modifying substrate accessibility and influencing fermentation trajectories. Unlike microbial-derived enzymes, which are dynamically produced according to microbial physiology and environmental conditions, externally supplied enzymes provide controllable modification of complex food matrices before or during fermentation. Their contribution extends beyond accelerating hydrolysis reactions because enzyme supplementation alters the availability and distribution of fermentable carbon and nitrogen sources, thereby potentially affecting microbial growth and subsequent metabolite formation. Therefore, exogenous enzymes should be considered targeted biochemical interventions that modify substrate availability and influence microbial responses rather than merely processing aids for improving degradation efficiency.

In plant-based and protein-rich systems, exogenous enzymes are frequently used to overcome structural limitations that restrict access to otherwise poorly available substrate fractions. Carbohydrases can increase the release of soluble carbohydrates from plant matrices, whereas proteolytic enzymes can generate peptides and amino acids from protein-rich substrates. For protein substrates, controlled enzymatic hydrolysis has been shown to modify protein structure and increase the production of lower-molecular-weight peptide fractions with altered functional properties (Christensen et al., 2022; Jin et al., 2016; Dabbour et al., 2020a,b, 2026; Dabbour et al., 2020a,b). These studies primarily establish biochemical effects of enzymatic pretreatment and should be distinguished from studies in which enzymatic intervention is directly integrated with subsequent microbial fermentation. In lactic-acetic acid co-fermentation of jujube puree, cellulase pretreatment combined with *Lactiplantibacillus plantarum* strain CGMCC 3346 and *Acetobacter pasteurianus* affected physicochemical characteristics, phytochemical composition, volatile profiles, antioxidant activity, and total phenolic content (351.89 mg GAE/100 g fresh weight) under optimized conditions (Boasiako et al., 2024a,b). This type of combined intervention more directly illustrates how enzyme-mediated matrix modification can be coupled to microbial conversion, although the relative contribution of cellulase activity and microbial metabolism to individual product characteristics remains difficult to separate. Similarly, enzyme-assisted processing of protein-rich substrates can increase peptide accessibility before or during microbial conversion (Hao et al., 2026). Accordingly, exogenous enzyme supplementation should be evaluated not only by the extent of hydrolysis, but also by the extent to which enzyme-generated products enter subsequent microbial metabolic pathways.

The ecological consequences of exogenous enzyme supplementation are influenced by the interaction among enzyme specificity, substrate structure, microbial utilization capacity, and fermentation conditions. Increased availability of soluble sugars, peptides, and amino acids may potentially alter microbial competition, cooperation, and metabolic flux distribution within fermentation communities. However, excessive hydrolysis may result in accumulation of intermediate metabolites or metabolic imbalance, whereas insufficient substrate modification may fail to overcome nutrient limitations. Therefore, the application of exogenous enzymes requires quantitative evaluation of enzyme dosage, substrate conversion kinetics, microbial responses, and final product characteristics. Although enzyme-assisted fermentation has demonstrated considerable potential, the mechanisms by which externally introduced enzymes reshape microbial ecological networks remain insufficiently understood.

### 2.3 Reciprocal interactions between enzymatic processes and microbial metabolic networks

The relationship between enzyme systems and microbial communities can be considered reciprocal because biochemical transformation modifies the resources available to microorganisms, while microbial growth and metabolism continuously alter the conditions under which enzymatic reactions occur. Enzymes convert complex substrates into more accessible compounds, whereas microorganisms influence enzymatic processes through changes in substrate consumption, enzyme production, pH, redox conditions, oxygen availability, and metabolite accumulation. Fermentation performance therefore emerges from iterative feedback between substrate transformation and microbial functional responses rather than from independent contributions of enzymes or microorganisms. Within this framework, enzyme-mediated resource release can modify microbial growth opportunities, while microbial physiological and environmental changes can in turn alter subsequent enzyme expression and catalytic performance. These reciprocal processes are summarized in Fig. 3.

**Fig. 3:**
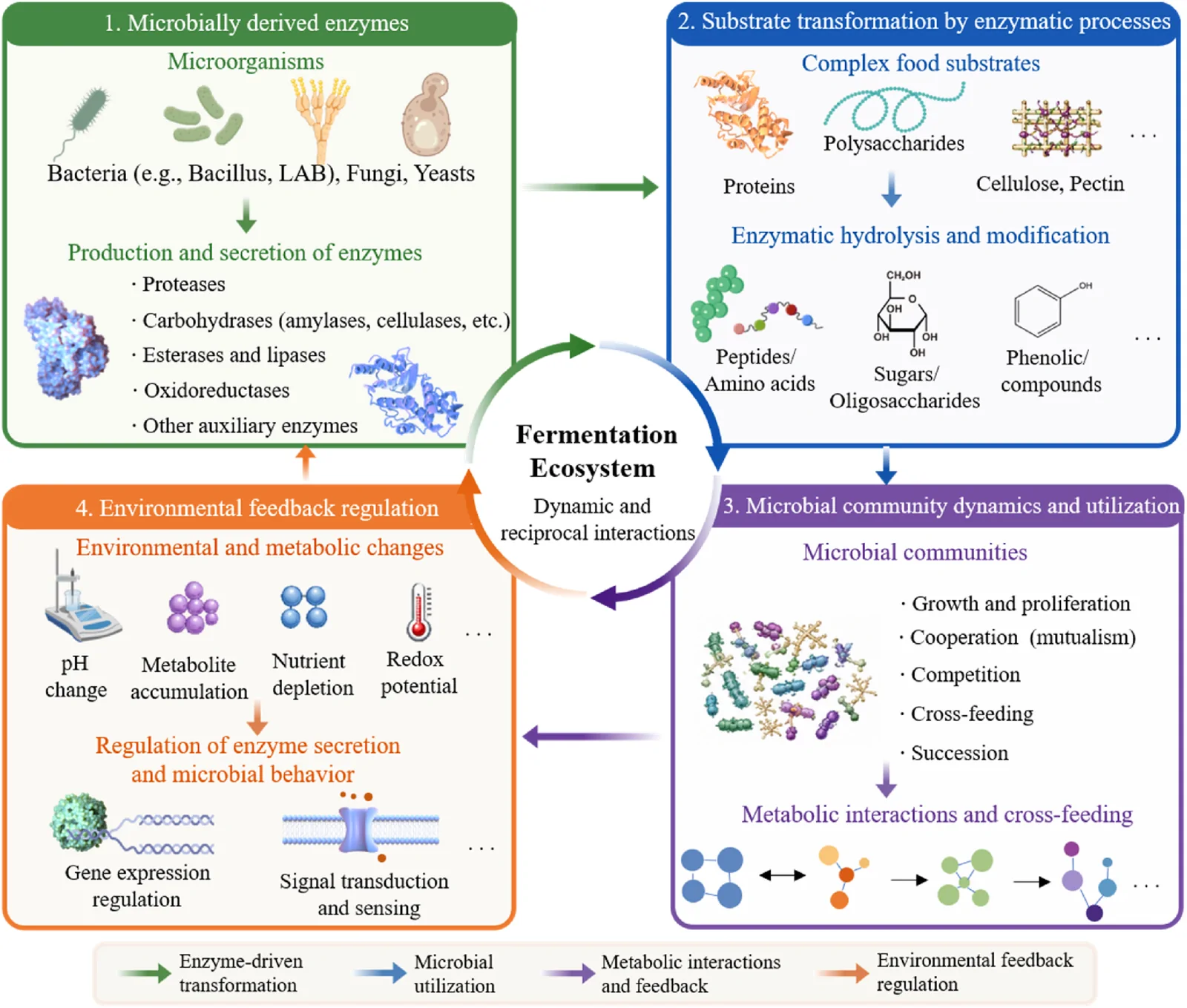
Feedback interactions between enzymatic processes and microbial community dynamics during fermentation.

Extracellular enzyme-mediated substrate depolymerization constitutes an important interface between biochemical transformation and microbial resource utilization. Degradation of polysaccharides, proteins, and other complex macromolecules generates soluble sugars, peptides, amino acids, and other low-molecular-weight intermediates that become available for microbial assimilation. The ecological consequence of this release depends on which microorganisms possess compatible transport and catabolic capacities, and on whether the released compounds are limiting, shared, or competitively consumed. Enzyme-mediated transformation can therefore alter resource distribution within fermentation ecosystems, but resource release alone does not establish that a specific ecological interaction or community shift has occurred. In enzyme-intensified microbial conversion systems, improved accessibility of recalcitrant substrates has been associated with enhanced microbial conversion efficiency, illustrating the functional value of coordinated enzymatic and microbial processing (Geng et al., 2024).

Microbial enzyme production is itself responsive to nutrient availability and environmental stress, creating a second level of reciprocity between biochemical transformation and microbial physiology. At the regulatory level, enzyme production and substrate-utilization pathways are controlled by nutrient-responsive and stress-responsive regulatory networks rather than by substrate availability alone. In many Gram-positive fermentative bacteria, carbon catabolite repression mediated by CcpA coordinates preferential utilization of readily metabolizable carbon sources and can suppress alternative carbohydrate-utilization pathways, whereas nitrogen-responsive regulators such as CodY couple intracellular amino-acid availability to proteolytic, transport, and biosynthetic functions. Acid stress, oxygen limitation, redox imbalance, and quorum-sensing signals can further modify transcriptional programs associated with extracellular enzyme production and metabolic adaptation. These regulatory systems therefore represent potential control points for mechanism-guided fermentation design; however, their effects are strongly strain- and environment-dependent, and direct validation in multispecies food fermentation communities remains limited. Changes in carbon and nitrogen availability can modify substrate utilization priorities, transport activity, and expression of catabolic functions, while acidification, oxygen limitation, redox changes, and nutrient depletion can further alter microbial metabolic state and enzyme production. In complex food fermentations, these regulatory responses are rarely resolved at the level of individual transcriptional regulators and are more often inferred from transcriptomic, proteomic, enzymatic, or metabolic changes. Consequently, specific regulatory mechanisms established in model microorganisms should not automatically be extrapolated to complex fermentation communities without system-specific validation.

Conversely, microbial succession can alter the enzymatic potential of a fermentation system because different populations differ in their capacities for enzyme production, substrate utilization, and environmental tolerance. At the same time, microbial metabolism modifies extracellular pH, redox state, oxygen availability, and nutrient composition, which can affect the activity and stability of enzymes already present in the system. Protease-assisted mixed fermentation of bean dregs and soybean meal provides an example in which exogenous proteolytic intervention was combined with a multi-strain fermentation system to improve substrate conversion and product characteristics (Heng et al., 2022). However, such combined-process studies do not necessarily resolve whether the observed effects arise from direct enzyme-microbe interactions, additive contributions, or secondary changes in substrate composition. Taken together, available studies support the view that enzymatic processes and microbial functions are interconnected within fermentation ecosystems, although evidence strength varies across catalytic, physiological, ecological, and community levels. Direct mechanistic understanding remains strongest in purified-enzyme, single-strain, and simplified co-culture systems, whereas causal validation in complex food matrices remains comparatively limited. Future studies should therefore establish quantitative relationships among enzyme abundance and activity, substrate conversion kinetics, microbial population responses, and metabolic flux redistribution through integrated transcriptomic or proteomic analysis, direct enzyme assays, targeted metabolite quantification, stable-isotope tracing, and controlled community reconstruction.

## 3 Microbial ecological dynamics and functional interactions in fermentation ecosystems

Microbial communities in fermentation ecosystems represent dynamic assemblages in which microorganisms interact with substrates, enzymes, metabolites, and environmental conditions to influence fermentation performance. Rather than being passive responders to enzyme-mediated substrate transformation, microbial populations actively participate in resource utilization, metabolic exchange, and community succession through their functional traits. Therefore, fermentation characteristics are influenced not only by microbial composition but also by functional organization, metabolic compatibility, and ecological interactions within microbial communities. During fermentation, microbial community structures continuously change in response to substrate availability, environmental conditions, and metabolite accumulation. These changes reflect adaptive ecological processes involving alterations in metabolic activity, enzyme production, and resource utilization strategies, which ultimately influence fermentation efficiency and product quality (Dai et al., 2023; Hu et al., 2023; Liu et al., 2024).

### 3.1 Microbial succession and functional differentiation

Microbial succession is a fundamental ecological process that shapes the temporal development of fermentation communities. During fermentation, microorganisms with distinct metabolic capabilities are selectively enriched according to environmental conditions, substrate characteristics, and interspecies interactions. In many fermentation systems, early colonizing microorganisms often contribute to initial substrate degradation through extracellular enzyme production, whereas subsequent microbial populations utilize the resulting metabolites for further biochemical conversion. Accordingly, microbial succession should be regarded not merely as temporal variation in community composition, but as progressive functional differentiation in which metabolic activities, enzyme production, ecological adaptation, and resource utilization are continuously reorganized throughout fermentation.

Recent studies have demonstrated that microbial succession is frequently accompanied by changes in metabolite profiles, nutritional characteristics, and sensory attributes of fermented foods. In soybean meal fermentation, for example, temporal variation in bacterial community composition occurred together with changes in peptide accumulation and metabolite composition (Liu et al., 2023). Although these associations suggest functional coupling between microbial dynamics and biochemical transformation, they do not independently establish that changes in community composition are the primary drivers of the observed metabolic outcomes. Moreover, microbial abundance alone is insufficient to estimate ecological importance because microorganisms present at relatively low abundance may contribute disproportionately through specialized enzymatic functions or metabolically important interactions. Therefore, interpretation of fermentation ecology should integrate microbial abundance with functional traits, including substrate utilization capacity, enzyme production, stress tolerance, and experimentally supported metabolic activity.

Environmental factors further regulate microbial succession by modifying ecological niches and metabolic constraints. Temperature, pH, moisture, oxygen availability, and substrate composition influence microbial selection and functional differentiation by affecting growth advantages and metabolic limitations. For example, temperature variation has been shown to reshape microbial community composition and volatile compound formation during fermentation (Dai et al., 2023). Collectively, these observations support the interpretation that microbial succession reflects ecological adaptation under changing fermentation environments rather than a predetermined sequence of taxonomic replacement.

### 3.2 Microbial cooperation, competition, and metabolic exchange

Microbial interactions contribute substantially to the stability and functionality of fermentation ecosystems. Within complex fermentation communities, microorganisms interact through combinations of cooperation, competition, metabolite exchange, environmental modification, and resource partitioning. Differences in substrate utilization, enzymatic capability, stress tolerance, and metabolic specialization create opportunities for both complementary and competitive ecological relationships (Wang et al., 2026a; Wu et al., 2026). Consequently, microbial interactions influence resource allocation, metabolic pathway utilization, and community development throughout fermentation.

Cooperative interactions may arise when microorganisms perform complementary metabolic functions that collectively improve substrate conversion. Mixed starter cultures containing lactic acid bacteria (LAB), yeasts, or other functional microorganisms have frequently been associated with improved flavor formation, nutritional characteristics, or fermentation efficiency (Gao et al., 2019; Xia et al., 2019). In many cases, such cooperation is facilitated through metabolite exchange, in which metabolites released by one microorganism become substrates for another population. Nevertheless, microbial cooperation should not be regarded as the default ecological state of fermentation ecosystems. Competition for nutrients, oxygen, micronutrients, attachment sites, and environmental niches occurs simultaneously and can strongly influence community composition and fermentation trajectories. Consequently, observed fermentation performance generally reflects the balance between complementary metabolism and competitive resource acquisition rather than cooperation alone.

Metabolic cross-feeding provides a representative mechanism underlying microbial cooperation in fermentation ecosystems, although its occurrence depends strongly on microbial compatibility and environmental conditions. In yeast–LAB fermentations, yeasts can release growth-associated metabolites, including amino acids, peptides, and B-group vitamins, which may alleviate nutritional limitations of LAB and support their proliferation. Conversely, LAB-derived organic acids, particularly lactate, can modify environmental conditions and serve as substrates for secondary microbial conversion. For example, in mixed fermentations containing LAB and acetic acid bacteria, lactate produced through LAB carbohydrate metabolism can be oxidized into acetate by acetic acid bacteria, representing a sequential carbon-transfer pathway. However, these interactions should not be inferred solely from microbial abundance patterns or metabolite correlations. Strong evidence for cross-feeding requires experimental validation through approaches such as mono-culture versus co-culture comparisons, metabolite supplementation or depletion experiments, strain omission/add-back experiments, or stable-isotope tracing.

The occurrence and quantitative importance of cross-feeding depend strongly on microbial species, strain combinations, substrate composition, inoculation strategy, and fermentation conditions. Consequently, cross-feeding should be interpreted as a context-dependent ecological mechanism rather than a universal characteristic of mixed fermentations. Importantly, the strength of evidence for cross-feeding depends on experimental design. Directional metabolite exchange is most convincingly supported by comparisons between mono-culture and defined co-culture systems, metabolite add-back or depletion experiments, strain omission and reconstitution, transporter or pathway perturbation, and stable-isotope tracing. In contrast, correlations between microbial relative abundance and metabolite concentrations provide only associative evidence and cannot independently establish metabolite transfer between community members.

In contrast, competitive interactions can influence community composition by limiting the proliferation of certain microorganisms and shaping ecological balance. Competition for nutrients, space, and environmental niches influences the relative abundance and activity of microbial populations during fermentation. The functional stability of fermentation ecosystems therefore depends on a dynamic balance among cooperation, competition, and metabolic complementarity rather than on cooperative interactions alone. Cross-feeding has been proposed as a mechanism contributing to metabolic complementarity and coexistence in certain microbial communities (Huang et al., 2025). However, ecological interactions remain highly context dependent. Differences in microbial species or strains, inoculation ratios, substrate composition, pH dynamics, oxygen availability, fermentation scale, and experimental design can substantially influence ecological outcomes. Accordingly, cooperation, competition, and metabolic exchange should be viewed as interacting ecological processes whose relative importance varies among fermentation systems rather than as fixed properties of microbial communities.

### 3.3 Synthetic microbial communities for controlled fermentation

The complexity and variability of natural fermentation ecosystems have stimulated increasing interest in the development of synthetic microbial communities (SynComs) with defined functional characteristics. Nevertheless, the current evidence supporting SynComs remains predominantly derived from laboratory-scale experiments, including defined co-culture systems and simplified model substrates. Although these studies provide important mechanistic information regarding metabolite exchange, division of labor, and community stability, their transferability to complex food matrices remains uncertain. The functional performance observed in simplified systems may not fully represent fermentation environments containing diverse substrates, spatial heterogeneity, indigenous microorganisms, and dynamic physicochemical changes. Therefore, evidence obtained from laboratory-designed consortia should be distinguished from validation in real food fermentation systems and pilot-scale applications.

Differences in substrate composition, microbial inoculation ratios, water activity, pH, temperature, oxygen availability, and fermentation duration may substantially influence community behavior and final product formation. Therefore, future studies should prioritize validation of SynComs in realistic food environments and pilot-scale systems to establish whether laboratory-observed interactions can be reliably translated into industrial fermentation processes. Within this context, SynComs provide a rational strategy for complementing traditional fermentation approaches by enabling function-oriented microbial assembly, thereby improving fermentation reproducibility, enhancing targeted metabolite production, and facilitating more predictable fermentation performance (Nikoloudaki et al., 2024; Giles-Gómez et al., 2026). However, improved predictability requires not only the selection of microorganisms with complementary metabolic functions but also quantitative understanding of their ecological interactions, population stability, and responses to environmental fluctuations during large-scale fermentation.

The rational construction of SynComs requires comprehensive understanding of microbial functions, enzyme capabilities, and interspecies interactions. Candidate microorganisms may be selected according to their contributions to substrate degradation, acidification, flavor formation, bioactive compound production, and environmental adaptation. Rational community design therefore emphasizes the integration of complementary enzymatic activities and metabolic functions instead of optimizing individual microbial performance alone. Future advances may benefit from combining microbial community design with enzyme engineering, multi-omics characterization, and computational modeling to identify functional combinations that are both mechanistically interpretable and industrially applicable. Nevertheless, challenges related to long-term community stability, reproducibility across batches, regulatory requirements, and scalability remain important considerations for practical implementation.

## 4 Metabolic transformations mediated by enzyme–microbial interactions

Metabolic transformations during fermentation arise from coordinated interactions among enzymatic activities, microbial metabolism, substrate characteristics, and ecological processes operating within fermentation ecosystems. Rather than directly determining final fermentation products, enzyme-mediated reactions primarily influence the accessibility and composition of metabolic precursors, whereas microbial communities determine how these precursors are assimilated, transformed, or further metabolized under specific ecological conditions. Accordingly, fermentation metabolism should be regarded as a dynamic process involving substrate conversion, metabolic adaptation, and pathway allocation rather than a simple sequence of independent biochemical reactions.

Within enzyme–microbial synergistic fermentation systems, the same enzymatically generated intermediates may enter different metabolic pathways depending on microbial composition, ecological interactions, and environmental conditions. Microbial communities therefore influence metabolic allocation within fermentation ecosystems that determine whether available carbon and nitrogen resources are converted into organic acids, alcohols, volatile compounds, bioactive metabolites, or microbial biomass. Through these interactions, enzyme systems influence precursor availability and metabolic potential, while microbial activities further modify metabolic pathways through changes in substrate consumption, environmental conditions, and metabolic feedback (Wu et al., 2024; Song et al., 2022).

This perspective emphasizes that fermentation characteristics cannot be predicted solely from enzyme activity or microbial abundance. Instead, final product formation emerges from the coordinated regulation of enzyme-mediated precursor generation, microbial pathway selection, and ecosystem-level metabolic interactions. This integrated framework helps explain how coordinated carbohydrate transformation, protein conversion, and secondary metabolism collectively shape fermented food characteristics.

### 4.1 Enzyme–microbial interactions in carbohydrate transformation and carbon flux redistribution

Carbohydrate transformation represents a major metabolic process influenced by enzyme–microbial interactions during fermentation. The functional significance of carbohydrate-active enzymes (CAZymes) extends beyond polysaccharide degradation because their activities influence the availability and composition of carbon substrates accessible to microbial metabolism. Differences in enzyme specificity and substrate preference regulate the composition of released carbohydrates, thereby influencing microbial resource utilization patterns and subsequent carbon flux distribution. For example, starch-, cellulose-, hemicellulose-, and pectin-derived carbohydrates are converted into structurally diverse intermediates that may enter different microbial metabolic pathways depending on community composition and functional capacity. GH13 α-amylases, GH11 xylanases, GH3 β-glucosidases, and CE1 feruloyl esterases therefore contribute not only to substrate accessibility but also to changes in the composition and availability of carbon resources within fermentation ecosystem.

Genomic and functional analyses have demonstrated that microbial carbohydrate utilization capacity is associated with metabolic adaptation and fermentation performance. The presence of specific CAZyme repertoires enables microorganisms to occupy distinct ecological niches by determining their ability to access and transform different carbohydrate resources. *Lactiplantibacillus plantarum* strain 84-3 contains diverse CAZyme families, including GH1, GH13, GT2, and GT4, suggesting potential capacity for carbohydrate utilization during fermentation (Liang et al., 2023). Similarly, *Leuconostoc citreum* SH12 possesses 59 CAZyme-related genes involved in carbohydrate metabolism, potentially supporting utilization of multiple carbohydrates including glucose, sucrose, fructose, maltose, xylose, arabinose, mannose, and cellobiose (Dai et al., 2024). These results suggest that microbial carbohydrate utilization depends on both microbial metabolic capacity and substrate availability.

Once released, fermentable sugars become available to microbial populations with different metabolic capacities and ecological interactions, thereby shaping carbon-flux distribution and final metabolite profiles. In plant-based fermentation systems, enzymatic modification of structural polysaccharides may enhance carbohydrate and phytochemical accessibility, which can influence subsequent fermentation characteristics. For example, studies of plant-derived fermentation systems have shown that microbial activity and substrate transformation jointly contribute to changes in phytochemical composition and volatile profiles (Tchabo et al., 2017). Beyond supplying energy, carbohydrate transformation can also generate oligosaccharides and other intermediates with potential functional properties that undergo further microbial conversion. Therefore, the metabolic consequences of carbohydrate degradation are determined by the interaction among enzyme specificity, substrate structure, microbial metabolic capacity, and downstream carbon allocation rather than by hydrolytic activity alone.

### 4.2 Enzyme-mediated protein conversion and nitrogen metabolism

Protein transformation is another major metabolic process influenced by enzyme–microbial interactions, particularly in fermentation systems based on protein-rich substrates. Before microbial assimilation, enzymatic hydrolysis determines the accessibility and composition of nitrogen-containing substrates. Studies on rapeseed meal proteins have demonstrated that controlled proteolysis can generate peptide fractions with antioxidant potential, highlighting the potential of enzymatic processing to improve the functional properties of protein-derived substrates (Zhou et al., 2016). The conversion of food proteins into microbial-accessible nitrogen sources is mediated by a coordinated extracellular proteolytic system consisting of endoproteases, exopeptidases, and specific peptide-degrading enzymes. Microorganisms including *Bacillus* species, LAB, and filamentous fungi produce diverse extracellular proteases that initiate protein hydrolysis, while peptidases such as PepN and PepX further hydrolyze oligopeptides into amino acids that can be assimilated by microbial communities. This sequential proteolytic process regulates not only the extent of macromolecular degradation but also the composition and accessibility of the nitrogen pool available for microbial growth and metabolism. Therefore, proteolytic activity influences fermentation outcomes not simply through increased protein degradation, but through regulation of nitrogen availability and redistribution among microbial populations with different assimilation capacities. The released amino acids can subsequently enter microbial catabolic pathways associated with volatile compound formation, including higher alcohols and aldehydes. In certain LAB, glutamate metabolism can also be redirected toward γ-aminobutyric acid (GABA) production through glutamate decarboxylase GadB, representing an additional pathway linking protein degradation with functional metabolite formation.

Quantitative studies have demonstrated the contribution of microbial proteolytic systems to fermentation performance. During enhanced SSF of soybean meal with *Bacillus subtilis* strain SBM_1, *Bacillus subtilis* SBM_1 fermentation enhanced protease activity, increased peptide production, and reduced antinutritional factors in soybean meal fermentation (Liu et al., 2024). This example demonstrates that microbial proteolytic activity can modify the availability and molecular distribution of nitrogen-containing substrates, thereby altering the biochemical resource environment available for subsequent microbial metabolism.

Nitrogen metabolism during fermentation may involve interactions among extracellular hydrolysis, intracellular assimilation, and metabolite exchange. Proteolytic microorganisms initiate protein degradation by releasing peptides and amino acids, which can subsequently serve as substrates for other microorganisms within the community. Such metabolic exchanges contribute to microbial coexistence and functional differentiation. Controlled enzymatic hydrolysis has been applied to improve peptide release and modify protein-derived functional properties, whereas subsequent microbial transformation determines the metabolic fate of released nitrogen sources and fermentation-derived compounds (Dabbour et al., 2019, 2020a,b; Zhou et al., 2017). However, the fermentation outcomes of protein conversion are determined by the integration of enzymatic hydrolysis, microbial nitrogen assimilation, and subsequent metabolic transformation rather than by proteolytic activity alone.

### 4.3 Formation of flavor and bioactive metabolites through integrated metabolic pathways

The formation of flavor compounds and bioactive metabolites represents the final outcome of coordinated enzymatic reactions and microbial metabolic activities during fermentation. These metabolites arise from the redistribution of carbohydrate- and protein-derived precursors through specific microbial pathways rather than from enzyme availability alone. In yeasts, higher alcohols and esters are mainly generated through the Ehrlich pathway, in which amino acids released from enzymatic protein hydrolysis are converted into α-keto acids, aldehydes, and higher alcohols through sequential transamination, decarboxylation, and reduction reactions. Therefore, proteolytic activity indirectly influences aroma formation by controlling precursor availability. In LAB, heterofermentative metabolic routes regulate the production of compounds such as diacetyl and acetoin through pyruvate conversion pathways. In addition, amino acids released during fermentation may participate in Strecker degradation and subsequent Maillard reactions, generating aldehydes and other volatile compounds associated with complex fermented food aromas.

Enzymes participate in flavor development by converting precursor molecules, whereas microorganisms influence the subsequent metabolic fate of these compounds through diverse biochemical pathways (He et al., 2016; Kwaw et al., 2018a,b). Enzymes such as esterases, alcohol dehydrogenases, and oxidoreductases further contribute to the transformation of volatile precursors and intermediate metabolites, thereby affecting aroma complexity. However, the contribution of individual pathways to sensory outcomes depends on precursor availability, microbial metabolic activity, and compound-specific sensory thresholds (Boasiako et al., 2024a,b). Therefore, changes in metabolite abundance should not be directly interpreted as equivalent changes in sensory perception without considering quantitative measurements such as absolute concentration and odor activity values (OAVs).

The production of bioactive metabolites follows similar principles, where enzyme-mediated substrate release provides precursors and microbial metabolism determines their conversion into functional compounds. Peptides, organic acids, phenolic derivatives, and other secondary metabolites may contribute to antioxidant, antimicrobial, or potential functional properties of fermented foods. Nevertheless, the biological significance of these compounds depends on their chemical forms, bioavailability, and functional validation, which remain variable among different fermentation systems.

Overall, metabolic transformation in fermentation ecosystems is governed by the coordinated regulation of enzyme-mediated substrate conversion and microbial pathway selection. Current studies have identified major enzymatic reactions and metabolic routes involved in fermentation quality formation; however, predicting final product characteristics from enzyme activity and microbial composition remains challenging because metabolic flux is influenced by multiple interacting ecological and environmental factors. Integrating quantitative metabolomics, enzyme kinetics, and microbial functional analysis will be necessary to establish more reliable links between molecular mechanisms and fermentation outcomes.

## 5 Multi-omics and computational approaches for characterizing enzyme–microbial interactions in fermentation ecosystems

The complexity of fermentation ecosystems requires analytical strategies capable of characterizing relationships among microbial communities, enzyme systems, metabolic pathways, and fermentation outcomes. Because enzyme–microbial interactions involve multiple biological layers, conventional approaches based solely on microbial composition or final metabolite measurements are often insufficient to determine the mechanisms underlying fermentation performance. Recent advances in multi-omics technologies and computational approaches have therefore enabled characterization of fermentation systems by linking microbial composition, functional potential, expressed molecules, and metabolite profiles (Fig. 4).

**Fig. 4:**
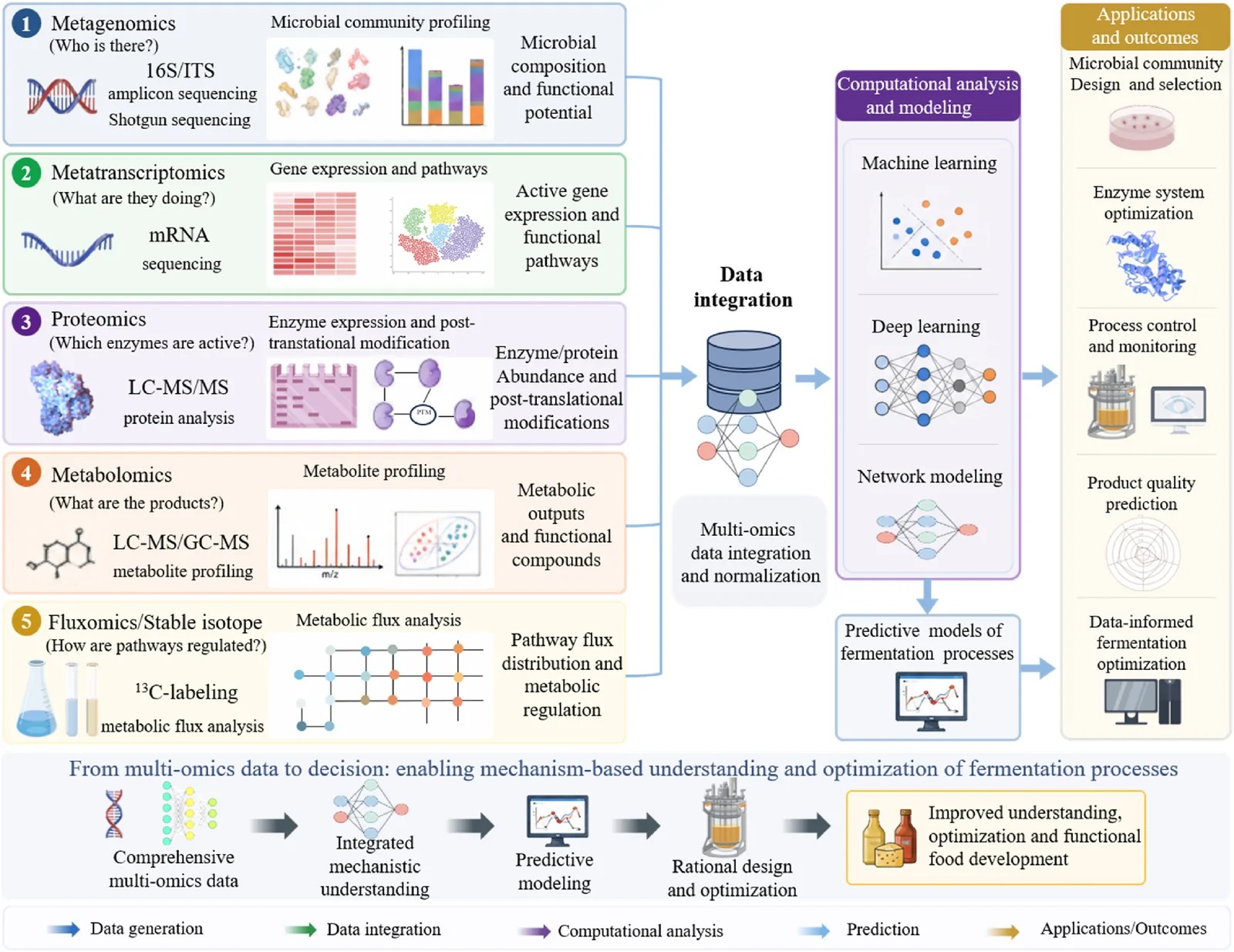
Multi-omics and computational approaches for characterizing enzyme–microbial interactions in fermentation ecosystems.

Unlike single-layer analyses, integrated multi-omics approaches provide complementary evidence for evaluating microbial functional potential, active expression patterns, and associations between enzymatic functions and metabolic outcomes. However, different omics datasets represent different levels of biological evidence and should not be interpreted as equivalent indicators of fermentation function. Metagenomics primarily reveals genetic potential, transcriptomics and proteomics provide evidence of functional expression, whereas metabolomics reflects the resulting biochemical consequences. Therefore, mechanistic interpretation of enzyme–microbial interactions requires integration across multiple omics layers rather than reliance on any single dataset.

Metagenomic analysis primarily reveals the genetic potential of microbial communities by identifying functional genes and predicted metabolic pathways, whereas metatranscriptomic and metaproteomic analyses provide evidence regarding actively expressed genes and translated functional proteins under specific fermentation conditions. Therefore, the presence of enzyme-related genes does not necessarily indicate active enzyme production or direct contribution to fermentation performance. Similarly, metabolomic profiles represent biochemical consequences of microbial activity but require integration with microbial and enzymatic information to determine the mechanisms responsible for metabolite formation. The major contributions of different omics approaches to deciphering enzyme–microbial interactions are summarized in Table 3.

**Table 3:** Multi-omics and complementary approaches for investigating enzyme–microbial interactions in fermentation systems.

Omics/computational approach	Information obtained	Application in enzyme–microbial fermentation studies	Current limitations	Recommended validation/interpretation	References
Metagenomics	Microbial composition, genome-level functional genes, and metabolic potential	Identifies populations carrying genes associated with carbohydrate degradation, proteolysis, transport, and stress adaptation	Reveals functional potential rather than active enzyme expression or catalytic activity	Integrate with transcriptomics, proteomics, enzyme assays, and metabolite measurements	(Zhao et al., 2015; Dai et al., 2024)
Metatranscriptomics	Condition-specific microbial gene-expression profiles	Evaluates transcriptional responses related to substrate utilization, enzyme-associated functions, and environmental adaptation	Transcript abundance does not necessarily reflect protein abundance or catalytic activity	Validate key responses at protein/activity level	(Zhao et al., 2019; Ji et al., 2025)
Metaproteomics	Protein and enzyme abundance; post-translational information	Detects expressed proteins potentially involved in substrate transformation, transport, and metabolic adaptation	Protein detection remains technically challenging and does not by itself establish catalytic activity	Combine with direct enzyme activity assays and substrate/product kinetics	(Zhao et al., 2015, 2019)
Untargeted metabolomics	Global small-molecule profiles	Identifies fermentation-associated changes in organic acids, peptides, phenolics, and volatile compounds	Metabolite variation alone cannot establish causal links to particular enzymes or microorganisms	Use for discovery, followed by targeted validation	(Zhang et al., 2019, 2022)
Targeted metabolomics	Absolute or validated quantitative measurement of predefined metabolites	Quantifies pathway-specific metabolites, functional compounds, and fermentation markers	Limited to predefined targets and dependent on prior pathway knowledge	Use internal standards and validated calibration wherever possible	(Zhao et al., 2021; Zhang et al., 2022)
Integrated multi-omics analysis	Combined information from microbial composition, transcripts, proteins, and metabolites	Links microbial functions, enzyme-related processes, and fermentation characteristics	Heterogeneous datasets, batch effects, different scales, and limited experimental validation complicate integration	Standardized metadata, harmonized preprocessing, and causal experimental validation	(Li et al., 2024; Ji et al., 2025)
Metabolic modeling and network analysis	Predicted pathways, interaction networks, and flux distributions	Explores possible metabolic exchanges and community-level metabolic organization	Predictions depend on model assumptions, annotation quality, and environmental constraints	Validate predicted exchanges by perturbation or isotope tracing	Karimi et al. (2026)
Artificial intelligence and machine learning	Data-driven relationships among multidimensional fermentation variables	Predicts fermentation performance and identifies influential process variables	Small datasets, overfitting, limited interpretability, and insufficient external validation remain common	Report prediction metrics, perform independent validation, and apply explainable methods such as SHAP where appropriate	(Ouyang et al., 2016; Xing et al., 2018; Wang et al., 2026b)

### 5.1 Metagenomics-based identification of microbial functional potential

Metagenomic sequencing provides an initial level of evidence for deciphering enzyme–microbial interactions by revealing the genetic potential of fermentation communities. Rather than directly measuring enzymatic activity, metagenomics identifies microbial populations carrying genes associated with substrate degradation, nutrient utilization, and metabolic adaptation, thereby defining the potential functional landscape of fermentation ecosystems. Compared with culture-dependent approaches, metagenomics enables comprehensive profiling of microbial diversity and functional gene repertoires, including genes encoding carbohydrate-active enzymes, proteases, transport systems, and stress-response pathways. Such information helps identify candidate microorganisms that may contribute to substrate transformation or potential ecological roles during fermentation.

For example, in thermophilic SSF systems, Hou et al. demonstrated that dynamic changes in microbial community composition were accompanied by variations in fermentation properties, suggesting that community succession is associated with changing environmental conditions and fermentation properties (Hou et al., 2019). These observations illustrate that metagenomic analyses can identify candidate functional microorganisms and predict their potential ecological roles within fermentation systems, thereby providing a valuable framework for subsequent mechanistic investigation.

However, the principal limitation of metagenomics is that it identifies functional potential rather than active biological function. The presence of genes encoding carbohydrate-active enzymes, proteases, transporters, or other metabolic proteins indicates that microorganisms possess the genetic capacity to perform corresponding reactions, but does not demonstrate that these genes are actively transcribed, translated, or contribute directly to fermentation performance under specific environmental conditions. Therefore, metagenomic analysis should be integrated with transcriptomic, proteomic, enzymatic, and metabolomic data to establish relationships between microbial identity, functional expression, and biochemical outcomes. This distinction is particularly important in enzyme–microbial fermentation systems because microorganisms possessing abundant enzyme-related genes may not represent the major contributors to substrate transformation, whereas low-abundance populations with highly expressed functional pathways may exert substantial ecological effects.

Collectively, metagenomics provides initial evidence for identifying potential microbial functions involved in enzyme–microbial systems by defining the functional potential of fermentation communities. Nevertheless, robust mechanistic interpretation requires subsequent validation at the levels of gene expression, protein production, enzyme activity, and metabolite formation, which are addressed in the following sections.

### 5.2 Transcriptomics and proteomics reveal active enzymatic functions

While metagenomics defines the functional potential of fermentation microbiomes, metatranscriptomic and proteomic analyses provide evidence regarding expressed biological processes. These approaches bridge the gap between predicted microbial capability and experimentally supported functional activity by revealing whether enzyme-related functions encoded in microbial genomes are actively regulated and expressed under specific fermentation conditions. Transcriptomic analysis identifies genes that are actively expressed under specific fermentation conditions, thereby revealing microbial physiological responses associated with substrate utilization, environmental adaptation, stress tolerance, enzyme secretion, and metabolite biosynthesis. Because transcription is highly dynamic and responds rapidly to changes in nutrient availability, environmental conditions, and microbial interactions, transcriptomic profiling provides important insight into the regulatory processes governing microbial adaptation throughout fermentation.

Nevertheless, transcript abundance should not be interpreted as direct evidence of functional enzyme activity. Gene expression is regulated by multiple post-transcriptional processes, and increased transcript levels do not necessarily result in proportional protein production or catalytic activity. Therefore, transcriptomic analyses primarily reveal regulatory responses rather than direct biochemical outcomes, and interpretation of enzyme-mediated functions requires complementary evidence from protein expression, enzymatic activity measurements, and metabolite production.

Proteomic analysis provides this additional level of validation by identifying proteins, including enzymes potentially involved in substrate transformation. Compared with transcriptomic data, proteomics more closely reflects the protein components associated with catalytic potential. In fermentation systems, proteomic analysis enables identification of enzymes and other functional proteins that are present or differentially abundant during carbohydrate degradation, protein hydrolysis, transport, stress adaptation, and secondary metabolism, although catalytic activity requires independent biochemical validation.

The integration of transcriptomic and proteomic analyses is particularly valuable for resolving regulatory mechanisms underlying enzyme–microbial interactions. For example, transcriptional activation of enzyme-related genes accompanied by increased abundance of corresponding proteins provides stronger evidence for functional regulation than either dataset alone. Conversely, discrepancies between transcript and protein levels may indicate post-transcriptional regulation, protein stability differences, or environmental constraints affecting enzyme activity. Integrated omics analyses have revealed relationships between microbial community composition, enzyme profiles, and metabolic adaptation, thereby improving mechanistic interpretation of fermentation processes (Zhao et al., 2019).

Collectively, transcriptomic and proteomic analyses provide the second level of evidence for interpreting enzyme–microbial interactions by identifying which microbial functions are actively expressed under specific fermentation conditions. However, neither transcript nor protein abundance alone can fully explain fermentation performance because the final biochemical outcome depends on enzyme activity, substrate accessibility, metabolic flux, and downstream metabolite formation. Therefore, integration with metabolomic analysis is required to connect active microbial functions with measurable fermentation phenotypes.

### 5.3 Metabolomics and metabolic modeling link biochemical activity with fermentation outcomes

Metabolomics provides direct measurements of biochemical changes associated with fermentation processes by characterizing the small molecules generated, transformed, and consumed throughout fermentation. Compared with metagenomic, transcriptomic, and proteomic analyses, metabolomics most directly provides information on metabolic outcomes because it captures the integrated biochemical consequences of gene expression, enzyme activity, and environmental adaptation. Therefore, metabolomic analysis provides an essential link between molecular mechanisms and fermentation phenotypes by revealing how enzyme-mediated substrate conversion and microbial metabolism are translated into measurable chemical outcomes.

However, the interpretation of metabolomic data depends strongly on analytical strategy and quantitative reliability. Untargeted metabolomics is primarily designed for comprehensive discovery of metabolic differences and pathway-associated changes, enabling broad characterization of fermentation-associated metabolic networks. Nevertheless, metabolite annotation confidence, quantitative reproducibility, and cross-platform comparability may vary substantially among analytical workflows. Targeted metabolomics complements untargeted approaches by providing more accurate quantification of predefined metabolites using validated analytical methods, thereby enabling more reliable evaluation of specific metabolic pathways and candidate biomarkers. Thus, untargeted and targeted metabolomics should be considered complementary strategies, with the former supporting metabolic discovery and the latter providing quantitative validation.

For volatile metabolites, relative peak intensity or normalized abundance alone should be interpreted cautiously because sensory perception depends not only on compound concentration but also on odor threshold. OAVs, calculated as the ratio between compound concentration and its sensory threshold, therefore provide a more biologically meaningful estimate of the potential sensory contribution of individual volatile compounds. Accordingly, absolute quantification, odor threshold information, OAV calculation, and sensory evaluation should be integrated when linking volatile profiles with perceived aroma characteristics. Such integration is particularly important because compounds showing high relative abundance do not necessarily represent the major contributors to sensory perception.

Metabolite profiles alone cannot determine the mechanisms responsible for metabolite accumulation because observed changes may reflect altered synthesis, degradation, transport, microbial cross-feeding, or redistribution among interconnected metabolic pathways. Therefore, interpretation of metabolomic data requires integration with microbial composition, enzyme activity measurements, and functional omics information to determine the biological processes driving metabolite formation. Metabolic flux analysis and computational metabolic modeling provide complementary approaches by interpreting metabolite changes in relation to biochemical pathways and identifying potential regulatory nodes associated with fermentation performance. Metabolic modeling approaches have been developed to integrate omics datasets and explore community-level metabolic networks and predict potential microorganisms, enzymes, and pathways associated with substrate conversion and product formation (Wang et al., 2026b).

Collectively, metabolomic analyses provide biochemical evidence linking enzyme activity with fermentation phenotypes, whereas metabolic modeling extends these observations by providing mechanistic interpretation of metabolic network organization. Together with genomic, transcriptomic, and proteomic evidence, these approaches establish a multi-level framework for understanding enzyme–microbial interactions and provide the biochemical basis for predictive modeling and intelligent fermentation optimization discussed in the following section.

### 5.4 Artificial intelligence-assisted prediction and optimization of fermentation processes

The integration of multi-omics datasets has increased the demand for computational approaches capable of identifying complex relationships among microbial functions, enzyme activities, and metabolic outcomes. Rather than replacing mechanistic investigation, AI serves primarily as a computational framework for integrating complex biological datasets and identifying nonlinear relationships among microbial communities, enzyme-related features, environmental variables, and metabolic outputs that are difficult to resolve using conventional statistical approaches. By combining multi-dimensional datasets, AI-assisted approaches have the potential to improve prediction of fermentation trajectories, identify key functional factors, and support process optimization. However, the reliability of AI-based predictions depends strongly on data quality, model selection, and independent validation (Ouyang et al., 2016; Xing et al., 2018; Zhao et al., 2025). Recent studies have further expanded these approaches by integrating spectroscopic monitoring, multi-omics information, and machine-learning algorithms for real-time fermentation prediction and process optimization.

Machine-learning approaches have been applied to predict fermentation characteristics, classify microbial community patterns, and establish relationships between process parameters and product quality (Dong et al., 2017; Ouyang et al., 2016). Representative studies also illustrate why model performance should be reported quantitatively rather than described only as satisfactory. In rice-wine fermentation, a synergy interval partial least-squares model yielded prediction-set correlation coefficients (*R*_p_) of 0.8694, 0.8097, and 0.9039 for total sugar, alcohol content, and pH, respectively, with corresponding *RMSEP* values of 0.438, 0.617, and 0.0805 (Ouyang et al., 2016). In solid-state fermentation of rapeseed meal, near-infrared spectroscopy combined with interval partial least squares achieved an *R*^2^ of 0.9441 and an *RMSECV* of 0.654 g/L for polypeptide prediction using 81 samples, and prediction performance was further examined using an external validation set (Xing et al., 2018). Importantly, model performance should be interpreted according to the validation strategy employed. Calibration statistics, such as R^2^c and RMSEC, mainly reflect the ability of a model to describe the training dataset, whereas prediction statistics, including *R*^2^_p_, *RMSEP*, and *RPD*, provide stronger evidence for predictive capability. However, even models showing satisfactory prediction performance may have limited applicability when independent external validation across different fermentation batches, raw-material sources, facilities, or production scales is lacking. Therefore, the current application of machine learning in fermentation remains constrained by small datasets, potential overfitting, insufficient cross-system validation, and limited standardization of input variables and metadata. Future development will require larger benchmark datasets, standardized data acquisition protocols, and interpretable modeling strategies that can identify biologically meaningful predictors rather than only optimize predictive accuracy. Models developed from specific fermentation batches, substrates, or facilities may not perform reliably when applied to independent datasets with different microbial compositions or environmental conditions. Therefore, external validation across different fermentation systems is essential for evaluating model robustness and practical applicability.

Interpretability represents another major challenge for applying AI in fermentation biotechnology. Although complex models can identify relationships among large numbers of variables, the biological meaning of these relationships may remain unclear without appropriate interpretation strategies. Approaches such as variable importance analysis, SHAP (Shapley additive explanations), and other explainable AI methods can help identify key microbial, enzymatic, or environmental features associated with model predictions. Such strategies are particularly important for enzyme–microbial fermentation systems because practical application requires not only accurate prediction but also mechanistic understanding of the factors controlling fermentation behavior.

The application of AI also depends on the standardization and integration of heterogeneous fermentation datasets. Current challenges include limited sample sizes, inconsistent metadata reporting, insufficient cross-batch validation, and differences among analytical platforms, which may reduce model transferability between fermentation systems. Differences in experimental design, analytical platforms, metadata reporting, and data processing procedures can introduce substantial variation and limit model transferability. Improved model transferability will require standardized metadata reporting, including adoption of community standards such as MIxS where applicable, deposition of sequencing and metabolomics datasets in accessible public repositories, harmonized preprocessing and normalization workflows, and explicit assessment and correction of batch effects. These measures are necessary to improve comparability among studies and facilitate development of robust predictive models.

Overall, AI-assisted fermentation design provides promising opportunities for integrating microbial ecology, enzymology, and process engineering. However, current applications should be considered as complementary tools that support, rather than replace, experimental validation and mechanistic investigation. Future advances will require integration of explainable AI, standardized multi-omics datasets, and experimentally validated biological knowledge to achieve reliable prediction and control of enzyme–microbial fermentation systems.

## 6 Applications of enzyme–microbial interaction strategies in food biotechnology

Enzyme–microbial interaction strategies have attracted increasing attention as mechanism-guided approaches for improving fermentation performance and developing fermented foods with enhanced nutritional, sensory, and functional characteristics. Compared with conventional fermentation optimization strategies primarily focusing on strain selection or process regulation, enzyme–microbial strategies attempt to regulate fermentation outcomes through coordinated manipulation of substrate transformation, microbial metabolism, and ecological interactions.

Current applications mainly involve three major directions: functional fermented food development, nutritional enhancement, and sustainable utilization of food resources. Although these applications involve different substrates and microbial systems, they share a common principle in which targeted modification of microbial functions or enzymatic activities is used to regulate fermentation performance. Representative applications of enzyme–microbial interaction strategies are summarized in Table 4.

**Table 4:** Representative applications of enzyme–microbial interaction strategies in food biotechnology.

Application area	Substrate/fermentation system	Enzyme–microbial strategy	Main reported outcomes	Evidence context	References
Plant-based fermented foods	Jujube puree and related fruit matrices	Cellulase-assisted substrate modification followed by LAB–AAB fermentation	Modified physicochemical characteristics, phytochemical profiles, volatile composition, and antioxidant-associated properties	Direct enzyme-assisted microbial fermentation in a food matrix	Boasiako et al. (2024b)
Protein-rich fermented foods	Soybean meal and other soybean-derived substrates	Protease-producing microorganisms combined with SSF	Increased protease activity, peptide accumulation, improved nitrogen accessibility, and reduction of antinutritional protein factors	Direct microbial-enzyme fermentation evidence	(Dai et al., 2017; Liu et al., 2024)
Plant protein bioconversion	Rapeseed meal and related protein-rich agricultural substrates	Proteolytic transformation integrated with microbial fermentation	Improved protein degradation and peptide generation, with downstream changes in substrate utilization	Combined biochemical and fermentation evidence	(Hou et al., 2019; Dai et al., 2022a,b)
Agricultural by-product valorization	Corncob and lignocellulosic biomass	CAZyme-rich microbial consortia coupled with enzyme-mediated structural-carbohydrate degradation	Enhanced lignocellulosic conversion and generation of fermentation-associated products	Consortium-level biomass fermentation evidence	Geng et al. (2024)
Plant-based functional beverages	Mulberry juice and fruit fermentation systems	Microbial fermentation affecting phenolic transformation and volatile formation	Changes in phenolic compounds, flavonoids, anthocyanins, antioxidant-associated properties, and sensory-relevant metabolites	Microbial-fermentation evidence; enzyme involvement should be stated only when directly measured	(Kwaw et al., 2017, 2018a,b)
Fermented cereal-based foods	Barley-based fermentation	LAB fermentation associated with transformation of cereal-derived compounds	Altered metabolite profiles, phytochemical composition, and potential functional properties	Microbial-fermentation evidence; not direct evidence of exogenous enzyme–microbe synergy	(Zhang et al., 2016, 2022)
Process monitoring and optimization	Solid-state and liquid fermentation processes	Integration of enzyme/activity analysis, spectroscopy, microbial characterization, and computational approaches	Improved monitoring of fermentation progression and process consistency	Process-level enabling strategy rather than direct interaction mechanism	(Zhu et al., 2016; Xing et al., 2018; Wang et al., 2026a)

### 6.1 Development of functional fermented foods

The development of fermented foods with enhanced nutritional, sensory, and bioactive characteristics represents a major application of enzyme–microbial interaction strategies. In functional fermented food production, enzymatic intervention may expand opportunities for modifying nutritional and bioactive characteristics of fermented products. The incorporation of targeted enzymatic interventions further expands these capabilities by modifying substrate structures, improving accessibility of nutritional components, and providing additional precursors for microbial transformation.

In plant-based fermentation systems, enzyme-mediated modification of food matrices may facilitate the release or transformation of phenolic compounds and other components that can influence subsequent fermentation characteristics. Rather than acting independently, enzymatic pretreatment and microbial bioconversion function sequentially, with enzyme-mediated substrate deconstruction creating the biochemical conditions required for microbial transformation into metabolites with altered bioavailability and biological activity. Microbial fermentation has been shown to modify phytochemical composition and antioxidant properties (Kwaw et al., 2017, 2018a,b). Enzyme-assisted strategies may further enhance substrate accessibility in specific systems. These findings indicate that improvements in functional properties may result from combined effects of enzymatic treatment and microbial fermentation.

Protein-rich fermented foods provide another representative application of this synergistic strategy. In soybean- and cereal-based fermentation systems, microbial-derived proteases together with exogenous proteolytic enzymes promote protein hydrolysis and generate peptides and amino acid derivatives that may subsequently participate in microbial metabolism. Studies on fermented soybean products have demonstrated enhanced protein utilization and increased accumulation of functional components following coordinated enzymatic and microbial transformation (Dai et al., 2017; Zhang et al., 2019). Such applications illustrate that enzyme–microbial strategies can improve fermented food quality by simultaneously regulating substrate conversion, microbial metabolism, and the generation of functional compounds.

However, the effectiveness of enzyme-assisted microbial fermentation remains dependent on substrate composition, microbial compatibility, enzyme specificity, and fermentation conditions. Therefore, future development of functional fermented foods should focus on establishing predictable relationships between enzymatic modification, microbial metabolic pathways, and final product characteristics rather than relying solely on empirical formulation.

### 6.2 Enhancement of nutritional and functional properties

An important application of enzyme–microbial interaction strategies lies in improving the nutritional availability and functional value of food components. Unlike conventional approaches that focus on increasing the content of individual nutrients or bioactive compounds, enzyme–microbial synergistic fermentation may improve nutritional characteristics through coordinated modification of food structures, formation and transformation of fermentation-derived metabolites. Many food matrices contain complex macromolecular structures and antinutritional factors that limit nutrient accessibility. Enzyme-mediated degradation increases the availability of fermentable substrates, whereas microbial metabolism further transforms these intermediates into compounds with improved nutritional or functional characteristics.

Protein-rich raw materials provide representative examples of this coordinated transformation. Microbial proteases facilitate protein hydrolysis and promote the release of peptides and amino acid derivatives, while subsequent microbial metabolism determines their conversion into nutritionally and functionally relevant products. Therefore, the nutritional outcome of protein fermentation depends not only on the degree of hydrolysis but also on the balance among enzyme specificity, peptide utilization, microbial nitrogen metabolism, and downstream metabolite formation. Enzymatic hydrolysis efficiency and microbial metabolic capacity jointly influence nutritional outcomes (Ding et al., 2019; Xing et al., 2018).

Comparable mechanisms operate during carbohydrate transformation. Enzymatic degradation of structural polysaccharides increases substrate accessibility and releases soluble carbohydrates together with associated phytochemicals, whereas microbial metabolism determines their subsequent conversion into fermentation-derived metabolites. Fermentation of cereal-based substrates has been shown to alter phytochemical composition and nutritional properties in specific systems (Xiao et al., 2020; Zhang et al., 2017). These findings indicate that nutritional enhancement results from the interaction among substrate transformation, microbial bioconversion, and metabolite redistribution rather than from increased enzyme activity or microbial growth alone.

In addition to improving nutrient accessibility, enzyme–microbial interactions contribute to nutritional enhancement through reduction of antinutritional factors and modification of bioactive compounds. Coordinated enzymatic degradation and microbial metabolism can convert complex food components into more accessible forms and generate fermentation-derived compounds with potential functional properties. However, the biological significance of these changes depends on compound structure, bioavailability, dose, and functional validation, which remain variable among different fermentation systems. Therefore, future studies should establish quantitative relationships among enzyme activity, microbial metabolic pathways, nutrient transformation, and physiological functionality to facilitate more predictable nutritional enhancement.

### 6.3 Sustainable utilization of food resources and bioprocess optimization

Beyond functional food development, enzyme–microbial interaction strategies may contribute to more efficient utilization of food resources. Among these applications, the valorization of agricultural and agro-industrial by-products represents a particularly important direction because many biomass resources contain substantial amounts of proteins, polysaccharides, dietary fibers, and bioactive compounds that remain underutilized due to structural complexity and limited accessibility. SSF of agricultural and agro-industrial by-products represents a representative application of this framework, where enzyme-mediated degradation of complex biomass components enhances substrate accessibility and supports microbial conversion toward value-added products.

Enzyme-assisted microbial transformation can be performed under relatively mild conditions in many food-processing applications. Enzyme–microbial synergistic fermentation has been explored as an approach for biomass valorization, thereby promoting the production of functional peptides, bioactive metabolites, fermentation-derived ingredients, and other value-added products. For example, In specific biomass fermentation systems, enzyme-enhanced microbial conversion has improved substrate degradation and product formation (Geng et al., 2024). These applications illustrate that the contribution of enzyme–microbial interactions extends beyond product improvement and includes more efficient conversion of underutilized food resources into value-added products.

Successful industrial implementation, however, requires more than efficient substrate conversion alone. Large-scale fermentation depends on robust process monitoring, reproducibility, and process control to ensure consistent product quality across different production batches and manufacturing environments. Hyperspectral imaging has been explored for fermentation monitoring (Zhu et al., 2016), while multi-omics and computational modeling provide complementary approaches for system-level analysis. Rather than representing independent technological objectives, these analytical approaches serve as enabling technologies that facilitate industrial translation of enzyme–microbial interaction strategies into scalable and controllable fermentation processes.

Future industrial fermentation systems may integrate enzyme engineering, rational microbial community design, systems biology, explainable artificial intelligence, and intelligent process control into unified production platforms (Fig. 5). Nevertheless, widespread industrial adoption will require continued evaluation of economic feasibility, process robustness, regulatory compliance, manufacturing reproducibility, and life-cycle sustainability. Addressing these challenges will be essential for translating enzyme–microbial interaction strategies from laboratory investigations into practical food biotechnology and establishing more sustainable, predictable, and industrially applicable fermentation systems.

**Fig. 5:**
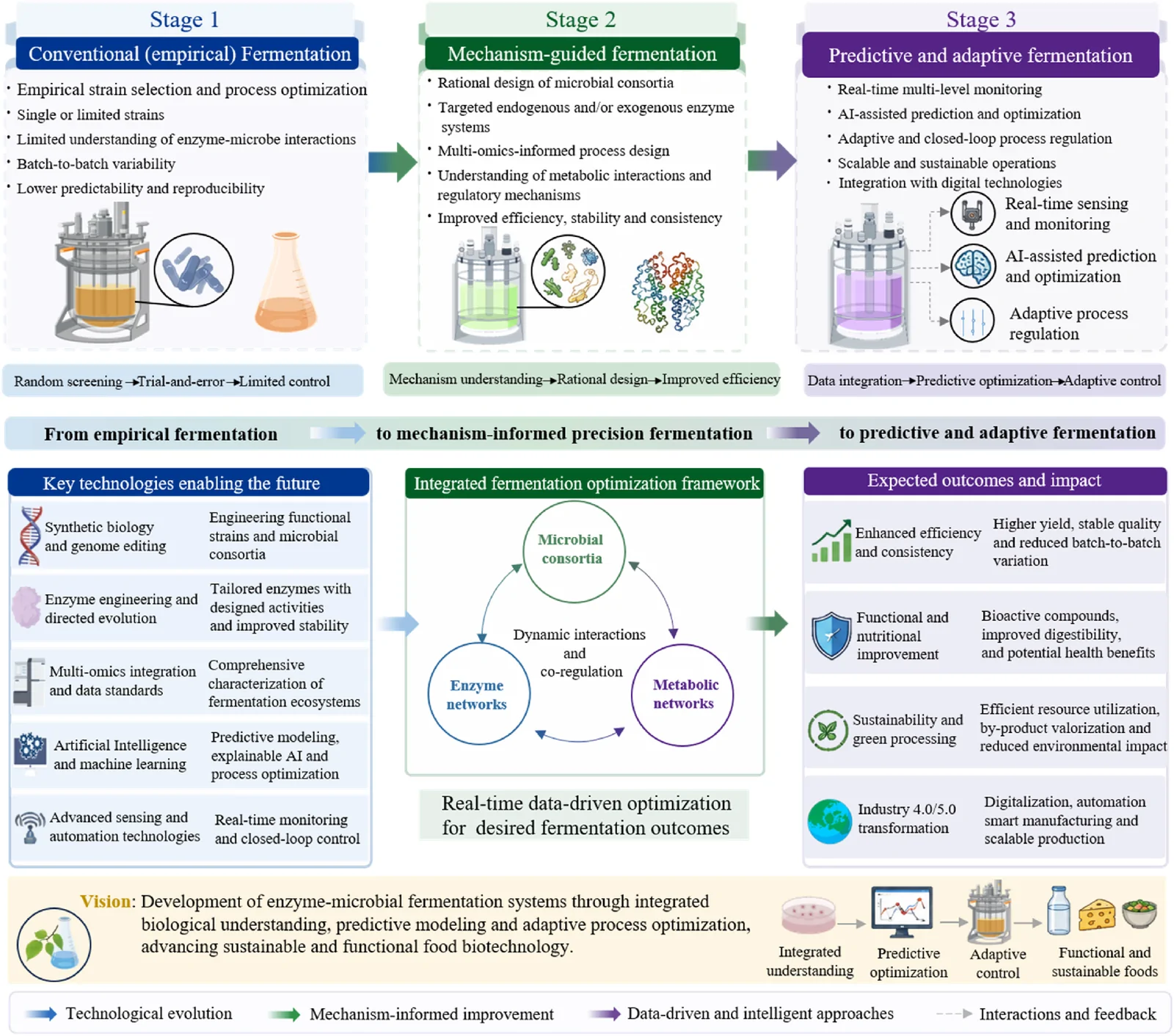
Future perspectives for improving predictability and controllability of enzyme–microbial fermentation processes.

## 7 Future perspectives for improving predictability and controllability of enzyme–microbial fermentation processes

Although increasing evidence suggests that enzyme–microbial interactions may influence fermentation characteristics, the major challenge for future development lies in translating emerging mechanistic insights into more predictable and controllable fermentation systems. Many fermentation processes still involve substantial empirical optimization, whereas quantitative relationships among enzyme activities, microbial ecological dynamics, metabolic flux, and product characteristics remain incompletely understood. Therefore, future progress should focus on establishing integrated frameworks that connect molecular mechanisms with microbial community design, process regulation, and predictive control. In this review, precision fermentation is used in a broader sense referring to mechanism-guided control of fermentation ecosystems for improving fermentation reproducibility, process robustness, and rational product design.

### 7.1 Toward more precise and mechanism-guided fermentation optimization

Conventional fermentation processes have traditionally been optimized through microbial strain selection, environmental regulation, and empirical adjustment of processing parameters. Although these approaches have enabled the successful production of diverse fermented foods, fermentation performance remains highly sensitive to variations in raw materials, microbial community composition, and environmental conditions, resulting in fluctuations in metabolite production and product quality.

In this review, precision fermentation is used in an extended sense to describe mechanism-guided and data-supported regulation of fermentation ecosystems rather than being restricted to engineered microbial production of defined molecules. Within the enzyme–microbial framework, this concept encompasses coordinated control of microbial functions, enzymatic activities, metabolic pathways, and environmental parameters to improve the predictability and reproducibility of fermentation outcomes. Its defining characteristic is therefore the transition from predominantly experience-based process adjustment toward mechanism-informed fermentation design and validation (Gao et al., 2025).

A major challenge remains the limited ability to predict microbial ecological responses under complex industrial fermentation conditions. Although multi-omics approaches provide increasingly detailed information regarding microbial functions and metabolic states, converting these data into reliable predictive models requires further integration of experimental validation, computational modeling, and quantitative analysis of microbial interactions.

### 7.2 Engineering microbial consortia for controlled fermentation

Natural fermentation ecosystems provide important ecological principles for engineering microbial consortia with complementary metabolic capabilities. Compared with single-strain fermentation systems, engineered microbial communities can potentially combine diverse functional traits, including substrate degradation, metabolite conversion, environmental adaptation, and stress tolerance, thereby providing greater flexibility for regulating fermentation performance.

Challenges associated with engineered microbial consortia include community stability, reproducibility, and scalability (Liu et al., 2025). Future development should therefore focus on improving our ability to characterize and predict ecological relationships. The long-term performance of engineered consortia depends not only on the presence of desired metabolic functions but also on population stability, resource allocation, interspecies interactions, and adaptation under changing fermentation conditions. Therefore, rational consortium design requires integration of microbial ecology, enzyme function, metabolic modeling, and experimental validation.

Advances in synthetic biology and metabolic engineering provide opportunities to construct microbial communities with defined functional characteristics. However, maintaining functional stability during prolonged fermentation remains a major challenge because microbial competition, evolutionary adaptation, environmental fluctuations, and scale-dependent effects can alter community composition and metabolic outputs. These factors may lead to differences between laboratory-designed communities and their performance in complex food matrices or industrial fermentation environments (Nikoloudaki et al., 2024).

Future research should therefore focus on identifying ecological principles governing microbial assembly, interaction networks, and community resilience. Combining synthetic microbial design with multi-omics monitoring and predictive modeling will be essential for developing engineered consortia that maintain functional stability and reproducible performance under realistic fermentation conditions. Such approaches will facilitate the transition from experimentally defined microbial communities toward robust and controllable fermentation platforms.

### 7.3 Intelligent monitoring and data-driven fermentation optimization

The integration of real-time sensing technologies, multi-omics analyses, computational modeling, and artificial intelligence provides opportunities for exploring fermentation monitoring and process optimization. Compared with conventional offline analysis, these approaches may enable more comprehensive assessment of microbial activity, enzyme production, metabolite accumulation, and fermentation progression (Yee et al., 2025; Adeleke et al., 2025).

Future intelligent fermentation systems should not rely solely on data-driven prediction but should integrate biological knowledge with real-time process information to achieve adaptive and interpretable control. Within enzyme–microbial fermentation systems, intelligent monitoring provides opportunities to connect microbial community dynamics, enzymatic activities, metabolic changes, and environmental parameters, thereby enabling more precise adjustment of fermentation conditions.

Artificial intelligence and machine-learning approaches provide valuable tools for identifying potential associations among microbial communities, enzyme activities, environmental parameters, and fermentation outcomes. However, practical implementation requires that predictive models are supported by reliable biological datasets and validated across different fermentation environments. Therefore, the future role of artificial intelligence may extend beyond prediction toward decision support, process optimization, and hypothesis generation for experimental validation.

Recent studies have demonstrated the feasibility of integrating multi-omics approaches with computational methods for fermentation analysis (Huang et al., 2025; Wang et al., 2026a). Additional technologies, including Raman spectroscopy and machine learning, have also been explored for fermentation monitoring and prediction. The current challenges and emerging strategies associated with industrial translation of enzyme–microbial fermentation systems are summarized in Table 5, including issues related to scalability, reproducibility, data integration, and process control.

**Table 5:** Current challenges, emerging strategies, and future priorities for industrial translation of enzyme–microbial synergistic fermentation systems.

Current challenge	Scientific basis of the limitation	Emerging strategies	Future priority for industrial translation	References
Incomplete mechanistic understanding of enzyme–microbial interactions	Quantitative relationships among enzyme activity, substrate accessibility, microbial ecological dynamics, metabolic flux distribution, and fermentation outcomes remain insufficiently resolved under complex food matrices	Integration of enzyme kinetics, stable-isotope tracing, direct enzyme assays, multi-omics datasets, controlled SynCom experiments, and mechanistic metabolic modeling	Establish quantitative causal relationships linking enzyme functions, microbial ecological interactions, metabolic allocation, and fermentation phenotypes across different fermentation system	(Wolfe and Dutton, 2015; Ji et al., 2025; Zhao et al., 2015)
Limited predictability and reproducibility of fermentation performance	Fermentation outcomes remain highly sensitive to raw-material variability, microbial composition, inoculation strategy, environmental parameters, and processing conditions	Standardized fermentation protocols, quantitative online monitoring, adaptive process regulation, and predictive computational models	Improve batch-to-batch consistency through mechanism-informed process control and standardized monitoring framework	(Hou et al., 2019; Tuly and Ma, 2024)
Difficulty translating laboratory-scale studies to industrial production	Most mechanistic studies are performed using simplified laboratory models rather than realistic food matrices or industrial-scale fermentations	Pilot-scale validation, food-matrix verification, scale-up optimization, and integration of real-time monitoring technologies	Develop robust enzyme-assisted microbial fermentation platforms that maintain functional stability across laboratory, pilot, and industrial production scales	(Geng et al., 2024; Zhu et al., 2016)
Limited ecological stability of synthetic microbial communities (SynComs)	Community composition may shift because of microbial competition, environmental adaptation, evolutionary dynamics, and functional drift during prolonged fermentation	Rational SynCom design based on metabolic complementarity, ecological stability, division of labor, and quantitative community monitoring	Establish predictable and resilient microbial consortia capable of maintaining long-term functional performance under industrial fermentation conditions	(Jin et al., 2024; Liu et al., 2025)
Challenges in integrating heterogeneous multi-omics datasets	Differences in sequencing platforms, analytical methods, metadata quality, batch effects, and data normalization complicate biological interpretation	Standardized metadata reporting (e.g., MIxS), harmonized analytical workflows, batch-effect correction, integrated computational pipelines, and public repositories	Facilitate reproducible systems-level interpretation of enzyme–microbial fermentation processes across independent studies and production platforms	(Li et al., 2024; Zhang et al., 2026b)
Limited interpretability of AI-assisted fermentation models	Machine-learning models often identify statistical associations without establishing biologically interpretable mechanisms or experimentally validated causal relationships	Explainable artificial intelligence (e.g., SHAP analysis), mechanistic metabolic modeling, causal inference, and experimental validation	Develop transparent decision-support systems that integrate predictive accuracy with mechanistic biological interpretation	(Ouyang et al., 2016; Xing et al., 2018; Zhao et al., 2025)
Insufficient real-time monitoring and adaptive process regulation	Conventional fermentation evaluation relies primarily on endpoint measurements, limiting timely intervention during dynamic fermentation processes	Integration of spectroscopy, biosensors, microbial functional analysis, AI-assisted feedback control, and adaptive fermentation regulation	Enable responsive process control through continuous sensing, intelligent prediction, and closed-loop optimization	(Zhu et al., 2016; Yee et al., 2025)
Regulatory, economic, and commercialization barriers	Large-scale implementation requires demonstration of production cost-effectiveness, microbial safety, regulatory compliance, consumer acceptance, and manufacturing reproducibility	Standardized evaluation frameworks, scalable enzyme production, validated microbial cultures, life-cycle assessment, and regulatory harmonization	Support commercialization of enzyme–microbial fermentation technologies through integrated technical, economic, environmental, and regulatory evaluation	(Gao et al., 2025; Pereira et al., 2025)

Nevertheless, a major challenge remains the establishment of biologically interpretable predictive frameworks capable of connecting microbial communities, enzyme systems, metabolic pathways, and product characteristics across multiple levels of biological organization. Future progress will therefore require coordinated development of standardized multi-omics resources, mechanistic metabolic models, explainable artificial intelligence, and experimental validation strategies to transform fermentation control from empirical adjustment toward more predictive and controllable process design.

## 8 Conclusion

In conclusion, enzyme–microbial interactions represent a critical regulatory layer linking substrate transformation, microbial ecological organization, metabolic redistribution, and fermentation outcomes. This review highlights that fermentation performance cannot be explained solely by microbial composition or enzyme activity, but instead emerges from the coordinated interactions among enzyme-mediated resource release, microbial functional traits, ecological relationships, and environmental adaptation. Within the proposed enzyme–microbial synergistic fermentation framework, extracellular enzymes function not only as catalysts for substrate degradation but also as regulators of microbial resource accessibility and metabolic opportunities, while microbial communities further influence enzymatic processes through succession, cross-feeding, competition, and environmental feedback. By integrating evidence from enzymology, microbial ecology, multi-omics, and computational approaches, this review provides a mechanistic perspective for understanding how molecular-level interactions contribute to fermentation phenotypes. The available evidence suggests that three principles are central to enzyme–microbial synergistic fermentation systems: enzyme-mediated substrate conversion determines the availability of carbon- and nitrogen-containing resources; microbial ecological interactions regulate the distribution and utilization of these resources; and integrated analytical approaches are required to connect functional potential, active biological processes, and measurable fermentation outcomes. Nevertheless, current understanding remains limited by insufficient causal validation in complex food matrices, variability among fermentation environments, and the lack of standardized datasets and predictive models. Addressing these challenges will require quantitative integration of enzyme activity, microbial functional traits, metabolic flux, and fermentation performance, together with improved validation of synthetic microbial consortia, genotype-guided strain selection, and cross-batch benchmark datasets. Such advances will facilitate the transition from empirical fermentation optimization toward more predictable, controllable, and mechanism-guided fermentation strategies.

## CRediT authorship contribution statement

Jing Hao: Conceptualization, Investigation, Methodology, Writing-original draft. Shengqi Zhang: Conceptualization, Investigation, Methodology. Feng Xiong: Conceptualization, Investigation, Methodology. Nan Wu: Conceptualization, Investigation, Methodology. Mokhtar Dabbour: Writing-review & editing, Methodology. Benjamin Kumah Mintah: Writing-review & editing, Methodology. Chunhua Dai: Methodology, Investigation. Rong Zhang: Methodology, Investigation. Zhaoli Zhang: Methodology, Investigation. Haile Ma: Validation, Conceptualization. Ronghai He: Conceptualization, Project administration, Supervision, Writing-review & editing.

## Declaration of competing interests

The authors declare that they have no known competing financial interests or personal relationships that could have appeared to influence the work reported in this paper.
